# ETSAM: Effectively Segmenting Cell Membranes in cryo-Electron Tomograms

**DOI:** 10.1101/2025.11.23.689996

**Published:** 2025-11-26

**Authors:** Joel Selvaraj, Jianlin Cheng

**Affiliations:** a Department of Electrical Engineering and Computer Science, University of Missouri, Columbia, 65211, MO, United States.;; b NextGen Precision Health, University of Missouri, Columbia, 65211, MO, United States

**Keywords:** Cryo-Electron Tomography, Cell Membrane, Segmentation, AI, Deep Learning

## Abstract

Cryogenic Electron Tomography (cryo-ET) is an emerging experimental technique to visualize cell structures and macromolecules in their native cellular environment. Accurate segmentation of cell structures in cryo-ET tomograms, such as cell membranes, is crucial to advance our understanding of cellular organization and function. However, several inherent limitations in cryo-ET tomograms, including the very low signal-to-noise ratio, missing wedge artifacts from limited tilt angles, and other noise artifacts, collectively hinder the reliable identification and delineation of these structures. In this study, we introduce ETSAM - a two-stage SAM2-based (1) fine-tuned AI model that effectively segments cell membranes in cryo-ET tomograms. It is trained on a diverse dataset comprising 83 experimental tomograms from the CryoET Data Portal (CDP) (2) database and 28 simulated tomograms generated using PolNet (3). ETSAM achieves state-of-the-art performance on an independent test set comprising 10 simulated tomograms and 15 experimental tomograms for which ground-truth annotations are available. It robustly segments cell membranes with high sensitivity and strong precision, achieving a more favorable precision–recall trade-off than other deep-learning methods. The ETSAM source code is freely available at https://github.com/jianlin-cheng/ETSAM.

Cryo-electron tomography (cryo-ET) has emerged as a powerful imaging technique for visualizing the three-dimensional (3D) architecture of biological specimens at near-atomic resolution in their native cellular environment. In cryo-ET, samples such as cells or tissues are rapidly frozen to preserve their native state. The frozen specimen is then imaged using a transmission electron microscope, where a series of two-dimensional (2D) projections is acquired by tilting the sample over a range of angles, typically ±60°. These projections are computationally reconstructed into a 3D volume, known as a tomogram, which provides detailed insights into subcellular structures, macromolecular assemblies, and dynamic cellular processes.

Cell membranes serve as dynamic barriers that compartmentalize cellular activities, facilitate signaling, and mediate interactions with the extracellular environment. Membranes play a vital role in many cellular functions such as vesicle trafficking (4, 5), synaptic transmission, conversion of energy in chloroplast thylakoids (6–10) and mitochondrial cristae (11–16), viral entry and budding (17–23), cell division (24, 25), organelle biogenesis (26), and the localization of membrane-associated proteins (27). Segmenting the membrane structures in cryo-ET tomograms enables researchers to identify membrane morphology (28) and curvature (29), thereby also understanding cellular function. For instance, precise membrane segmentation can help reveal how viruses like HIV (30, 31) or SARS-CoV-2 (19, 20) exploit host membranes during infection, or how defects in membrane architecture contribute to neurodegenerative diseases like Alzheimer’s (32, 33). Therefore, accurate membrane segmentation in cryo-ET tomograms has emerged as an important task in computational biology (34).

Traditional methods for membrane segmentation rely on manual or semi-automated approaches, often incorporating image processing techniques to enhance and delineate membrane features. One prominent example is TomoSegMemTV (35), which uses the scale-space operation based on Gaussian filtering to isolate features at a given membrane thickness, followed by the detection of ridge-like membrane regions via a Hessian tensor or edge-like membrane regions via a structure tensor. Further, it uses a tensor voting mechanism that allows voxels within the same membrane to reinforce one another, fill gaps, and disregard attached structures. However, it requires human intervention and parameter tuning to segment cell membranes from cryo-ET tomograms. Moreover, traditional approaches struggle with the variability in membrane appearance under different imaging conditions.

In recent years, deep learning methods have gained traction as promising alternatives for automating membrane segmentation in cryo-ET. Techniques such as Membrain-Seg (36) employ a U-Net-based model trained on annotated tomograms to predict membranes. It leverages the learned features to handle noise and variability more robustly than traditional methods. Similarly, TARDIS (Transformer-based Rapid Dimensionless Instance Segmentation) (37) uses a custom-designed Convolutional Neural Network (CNN) called FNet to identify and delineate cell membranes. TARDIS was trained on diverse annotated (labeled) tomograms. It also integrates a point-cloud-based DIST (Dimensionless Instance Segmentation) model for instance segmentation of predicted cell membranes. These deep learning-based approaches have demonstrated superior performance on benchmarks, reducing segmentation time from hours to minutes and enabling the scalable analysis of large cryo-ET datasets. However, membrane segmentation remains an open problem, as current deep learning models fail to achieve both high precision and recall without tradeoffs. Some models often miss critical membrane regions in their predictions due to low sensitivity, while other more sensitive models are prone to noisy predictions.

The challenges facing membrane segmentation in cryo-ET tomograms are largely due to their inherent limitations (38–40). Tomograms often suffer from a low signal-to-noise ratio (SNR) because electron doses must be minimized to prevent radiation damage to biological samples, resulting in noisy 3D reconstructions in which membrane boundaries are faint and difficult to distinguish from surrounding cytosolic or extracellular densities. Additionally, the missing wedge problem (41) arises from the limited tilt range during data acquisition, leading to incomplete sampling in the Fourier space and anisotropic resolution in the reconstructed tomogram. This manifests as elongation or blurring along the direction of the electron beam, distorting membrane shapes and complicating the identification of curved or closely apposed structures. Furthermore, sample preparation artifacts, such as ice contamination or uneven vitrification, introduce background scattering that diminishes membrane contrast and often creates crystalline patches or blobs that resemble membrane-like edges, leading to potential false positives in segmentation.

In this study, we introduce ETSAM, a fine-tuned two-stage Segment Anything Model (SAM2)-based (1) AI model for effectively segmenting cell membranes in cryo-ET tomograms. By repurposing SAM2’s video segmentation pipeline, ETSAM treats consecutive tomogram slices as a sequence of video frames. ETSAM leverages the SAM2’s memory encoder and memory attention mechanisms to effectively identify and track membranes across each slice of the tomogram. This approach enables the model to maintain consistency when tracking complex, curved membrane structures, while compensating for some of the inherent limitations of cryo-ET, such as low signal-to-noise ratio and missing wedge artifacts.

To train ETSAM, we collected 83 experimental tomograms from the CryoET Data Portal (CDP) (2) database along with their corresponding membrane annotations, and supplemented them with 28 simulated tomograms generated using PolNet (3). Since most membrane annotations from the CDP database are generated using AI tools with minimal human intervention, they often contain artifacts, false-positive predictions, or missing membrane regions. The selected 83 experimental tomograms are among the ones with minimal missing membrane regions in the CDP database. To further improve the quality, we reduced noise by manually cleaning the annotations. The first stage of ETSAM was extensively trained on all 111 tomograms in the training dataset. The fine-tuned first-stage ETSAM was then applied back to the training dataset to generate the membrane segmentation. The first-stage predicted membrane segmentation and the original tomograms of the training data were fused together as input to train the second stage of ETSAM, which outputs the final segmentation.

To evaluate ETSAM, we collected an independent test set comprising 15 experimental tomograms for which ground-truth annotations are available in the CDP database. We also supplemented the test set with 10 simulated tomograms generated using PolNet. ETSAM achieved state-of-the-art results on the test dataset compared to existing deep learning-based methods by attaining the highest Dice and Intersection over Union (IoU) scores. Additionally, ETSAM achieves a better precision-recall trade-off than the other methods by having a higher sensitivity to true positive membrane regions in the experimental tomograms while maintaining good precision.

## Results and Discussion

We evaluated ETSAM on the independent test dataset containing 25 tomograms. Dice, IoU, precision, and recall scores were used for assessing the quality of ETSAM-predicted membrane segmentation masks, and the average scores across the test tomograms are reported. Precision measures the accuracy of the model’s positive membrane predictions, whereas recall measures the completeness of it. Dice Score, also known as F1 score, is the harmonic mean of precision and recall, providing a single metric that balances both by weighting them equally. IoU score is the ratio of the area of intersection between the predicted segmentation and the ground truth to the area of their union, measuring the extent to which they overlap. ETSAM is compared with two other deep learning-based membrane segmentation methods: Membrain-Seg (36) and TARDIS (37). The default parameters are used for both methods. To evaluate the raw performance of each method, post-processing and test-time augmentations were not performed.

### Evaluation on Experimental Tomograms.

ETSAM was evaluated on 15 experimental tomograms from 6 cell datasets of 3 different cellular organisms. In each cell dataset, at most three tomograms were randomly selected to avoid bias from a single large cell dataset, resulting in a total of 15 test tomograms.

**Fig. 1.**
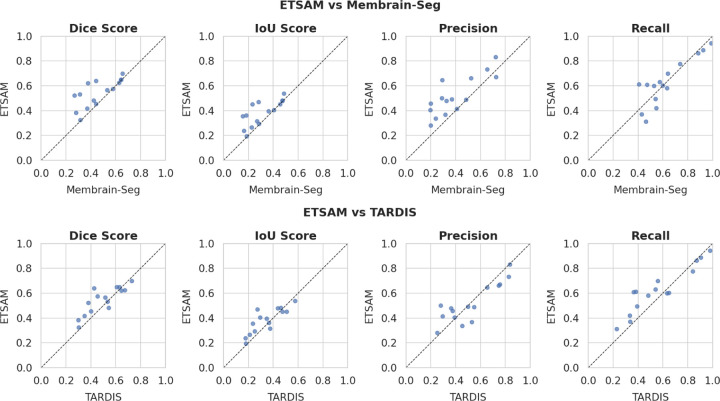
Per-tomogram performance of ETSAM on 15 experimental tomograms from the test dataset against Membrain-Seg and TARDIS in terms of multiple segmentation metrics.

As shown in Table 1, ETSAM delivers superior performance compared to Membrain-Seg and TARDIS, achieving the highest Dice score of 0.5427 and IoU score of 0.3796. This indicates a better overlap between predicted segmentation and ground-truth membrane regions. Additionally, ETSAM achieves the highest recall of 0.6269 and the second-highest precision of 0.5175, indicating that it captures the highest proportion of membrane regions with good precision. Membrain-Seg has the second-highest recall of 0.6245, but its precision is significantly lower at 0.3961. TARDIS’s precision of 0.5205 is slightly higher than ETSAM, but it has a substantially lower recall of 0.5661. Overall, ETSAM offers the best precision-recall trade-off, achieving the highest Dice, IoU, and recall scores while maintaining near state-of-the-art precision. This indicates that ETSAM can accurately predict more membrane regions while maintaining a relatively low level of false-positive predictions. Figure 1 and Supplementary Table S1 show the per-tomogram comparison of ETSAM, Membrain-Seg, and TARDIS. It can be observed that ETSAM consistently achieves better Dice and IoU scores across most experimental tomograms from different cell datasets.

**Fig. 2.**
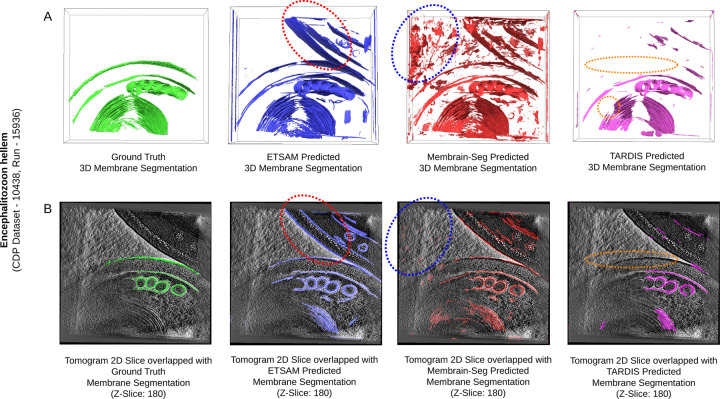
Visual comparison of the “ground-truth” cell membrane segmentation (green) of Encephalitozoon hellem (CDP Dataset - 10438, Run - 15936) against the ETSAM (blue), Membrain-Seg (red), and TARDIS (pink) predicted membrane segmentations. (A) Comparison of “ground truth” and predicted segmentation masks in three dimensions (3D). (B) The 2D tomogram slice (Z-Slice: 180) overlapped with the corresponding “ground truth” and predicted segmentation mask slices. Overlaying the 2D tomogram slice with the “ground truth” segmentation reveals that some true membranes in the top-right region of the tomogram slice are missed in the “ground truth” annotations but predicted by ETSAM (regions denoted by the red dotted ovals). The blue dotted ovals denote some false membrane regions over-predicted by Membrain-Seg, and the orange dotted ovals mark some true membrane regions missed by TARDIS.

It is worth noting that the “ground truth” segmentation of the experimental test tomograms were initially generated using AI tools and then adjusted by human annotators; they may contain errors, such as incomplete membrane segmentation, which may lead to the under-estimation of the performance of the methods. Figure 2 shows such an example, where the “ground truth” annotation includes the true membranes in the center and bottom but misses the true membranes in the top-right region that are predicted by ETSAM (red dotted oval region) and Membrain-Seg. Therefore, some true membrane regions were not treated as ground truth in the evaluation, leading to a lower precision score for them. Thus, a lower precision score can be attributed to either noisy segmentation predictions or missing ground truth annotations. Creating large datasets with more accurate and complete annotations is needed to further enhance the evaluation of membrane segmentation methods.

### Evaluation on Simulated Tomograms.

One way to overcome the limitation of incomplete ground-truth annotation is to evaluate the methods on simulated tomograms for which complete and accurate ground-truth membrane annotations are known. ETSAM, Membrain-Seg, and TARDIS were evaluated on the 10 simulated tomograms from the independent test dataset, which were generated using PolNet (3).

As observed in Table 1, ETSAM again leads in overall performance with the highest scores for Dice (0.6083), IoU (0.4385), and precision (0.5717). It also has the second-highest recall of 0.6612. Membrain-Seg has the highest recall of 0.7556 but scores a much lower precision of 0.4642. Both ETSAM and Membrain-Seg perform better on the simulated tomograms than on the experimental tomograms, whereas the opposite in the case of TARDIS. Among the three methods, TARDIS has the lowest scores on the simulated tomograms, different from its second-best overall performance on the experimental tomograms. TARDIS used CryoTomoSim (42) based simulated tomograms, whereas ETSAM and Membrain-Seg incorporated PolNet-based simulated tomograms as part of their training data. This can partially explain TARDIS’s low performance, as Membrain-Seg and ETSAM have seen similar types of PolNet simulated tomograms during the training process.

Although simulated tomograms with complete ground-truth annotations can be useful for further evaluating these methods, the results obtained from simulated tomograms alone should be interpreted with caution. Simulated tomograms may not perfectly replicate the diverse noise and signal patterns present in all experimental tomograms. Thus, a better performance on simulated tomograms can help attest a model’s overall performance in some situations, but lower scores on them do not necessarily indicate the model’s poor performance on experimental tomograms.

### Generalization to cryo-ET Tomograms of New Cellular Organisms.

To ensure that ETSAM can robustly segment cell membranes across a wide variety of cellular organisms, we also evaluated it on five additional experimental tomograms of different cellular organisms from the CDP database. The cellular organisms of these five experimental tomograms do not exist in the training data and have never been seen by ETSAM.

**Fig. 3.**
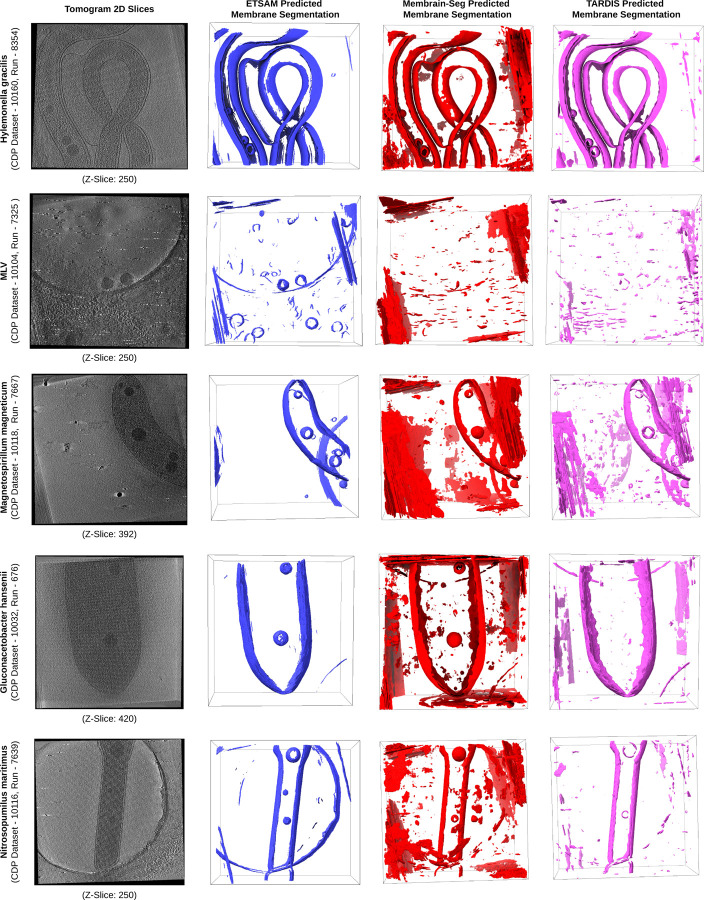
Visual comparison of ETSAM, Membrain-Seg, and TARDIS predicted membrane segmentation across 5 experimental tomograms of different cellular organisms unseen by ETSAM during its training. It appears that ETSAM predicts more true membranes (particularly some small ones) with less noise than the other two methods.

Figure 3 visualizes the ETSAM-predicted cell membrane segmentation of these experimental tomograms, along with their respective Membrain-Seg and TARDIS predictions. It can be observed that ETSAM can robustly segment cell membranes across the various cellular organisms. Moreover, in certain tomograms (CDP Runs - 7325, 7667, and 7639), ETSAM performs notably better than TARDIS or Membrain-Seg by predicting more cell membranes while generating less noise in the predicted segmentation. Although we cannot quantitatively evaluate ETSAM’s performance due to the lack of ground-truth annotations for these experimental tomograms, the visual comparison clearly validates that it generalizes effectively to these new cellular organisms that were never seen during training.

### Post-processing of Membrane Segmentation.

During prediction, ETSAM traverses each slice of the tomogram (similar to image frames in a video) to identify membrane boundaries, while its memory encoder and memory attention help track membrane regions across the slices. However, ETSAM may still predict a membrane-like region in a given slice but not observe a corresponding one in subsequent slices, which can lead to some false positive noise in membrane segmentation, thereby increasing the difficulty in visualizing and analyzing membrane regions.

To overcome this drawback, we developed a post-processing technique that can significantly reduce the visual artifacts in the segmented results. This is accomplished by identifying 3D blobs in the predicted segmentation mask and removing those that do not extend beyond a specific number (i.e., 10 in this experiment) consecutive slices. Because the size of noise blobs may vary across tomograms of different sizes, we provide users with an option to adjust the number of slices used to filter the blobs.

We have applied the post-processing technique to all tomograms in our test set, and the results are reported in Table 2. By comparing the ETSAM results before the post-processing (Table 1) and after the post-processing (Table 2), we can observe that post-processing slightly increases the dice, IoU, and precision scores due to the removal of small noises, while maintaining almost the same recall score. The most significant effect of the post-processing is to enhance the visual clarity of membrane segmentation by reducing noise, making it easy for users to analyze the membrane segmentation.

**Fig. 4.**
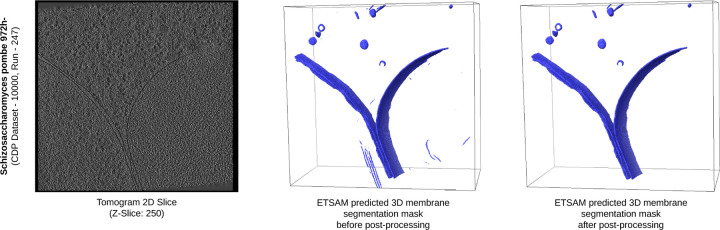
Visualization of the cryo-ET tomogram slice of Schizosaccharomyces pombe 972h- (CDP Dataset - 10000, Run - 247) along with ETSAM predicted 3D membrane segmentation before and after post-processing. Some noise predictions in the bottom region before post-processing are removed by the post-processing, while the true membranes are kept.

Figure 4 visualizes the tomography of Schizosaccharomyces pombe 972h- cell (CDP Dataset - 10000, Run - 247) and the ETSAM predicted segmentation before and after post-processing. It is observed that the post-processing technique removes most of the noisy regions while preserving the essential membranes predicted. In this particular tomogram, the post-processing of the ETSAM membrane segmentation improves the Dice score from 0.6987 to 0.6994, the IoU score from 0.5369 to 0.5378, and the precision from 0.8327 to 0.8350 with a negligible reduction in the recall score from 0.6018 to 0.6017. This demonstrates the effectiveness of the post-processing technique in removing noise while preserving biologically relevant membrane regions. Furthermore, Supplementary Figure S1 presents two additional examples of the visual improvements achieved by post-processing the segmentation of the experimental tomograms of Mus musculus cells.

While we recommend using the post-processing technique to improve the visual clarity of ETSAM membrane segmentation, caution must be exercised when dealing with thin and small membrane regions, as they may be removed during post-processing. In this case, we recommend visually comparing unprocessed and post-processed segmentation results to ensure biologically relevant membrane regions are not removed.

### Ablation Studies.

SAM2 (1) is a foundational AI image/video segmentation model that allows users to provide manual input prompts to segment and track regions of interest. However, we want ETSAM to operate in an automated manner, similar to other competing methods that require no manual user input during prediction. To address this problem, in our first ablation study, we tested three prompting techniques that automatically generate custom input prompts for ETSAM Stage 1 to segment and track all membrane regions effectively. The first prompting technique initializes an empty mask with zeroes in the first slice as the input prompt. The second prompting technique uses a mask-based prompt that is initialized with a grid of points (dotted grid pattern) spaced by 50 pixels in the XY plane of the first slice as the input prompt. The third prompting technique is similar to the second except that the dotted grid pattern is repeated in the first and every 50^*th*^ slice of the input prompt mask. For each pixel in a given tomogram slice, a logit value is predicted by the final layer of ETSAM’s mask decoder. The predicted logits can range from negative to positive values, with larger values indicating a higher likelihood of a cell membrane being present in that pixel. We used a default logit threshold of 0.0 for converting ETSAM Stage 1 predicted logits into binary segmentation masks, where any voxel with a logit value greater than the threshold is segmented as a cell membrane. These binary segmentation masks were then used for evaluation. ETSAM Stage 1 was evaluated using the three prompting techniques on the 15 experimental tomograms in the test dataset, and the results are tabulated in Supplementary Table S3. The first prompting technique of initializing an empty mask prompt performs poorly and fails to generate proper segmentation masks in some cases. The second prompting technique of initializing the grid of points at only the first slice achieves significantly better results than the other two approaches. This approach enables the first stage of ETSAM to concentrate equally on all regions initially, and as membrane regions are identified, it tracks them continuously using the memory features without interruption. This is unlike the third approach, where it is interrupted with a grid prompt at every 50^*th*^ slice and leads to poorer performance. Therefore, the second automated prompting technique that achieved the best results is selected as the default for Stage 1.

**Fig. 5.**
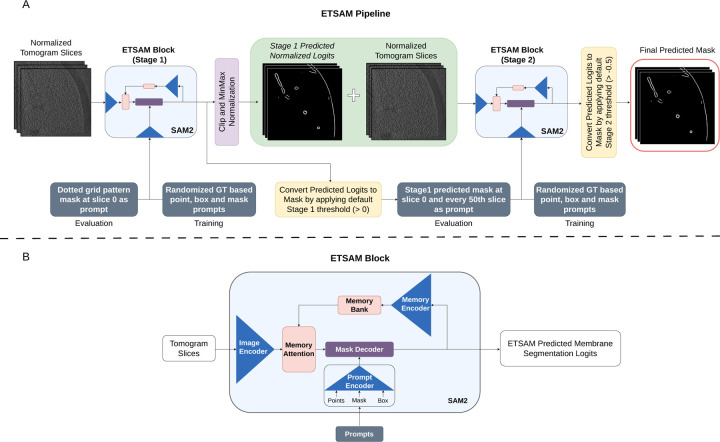
The workflow of the two-stage ETSAM. (A) The pipeline of the two-stage ETSAM. (B) The network architecture of the ETSAM block used in both stage 1 and stage 2.

In the two-stage ETSAM pipeline, as shown in Figure 5, the ETSAM Stage 1 predicted logits are clipped between −5 and 5, then min-max normalized to the 0 to 1 range, and added back to the original tomogram as input to ETSAM Stage 2. The ETSAM Stage 2 predicted logits are finally converted into a binary membrane segmentation mask using a logit threshold. In the ablation study of Stage 2 prediction, we conducted a comprehensive testing of various logit thresholds and prompting techniques. In addition to testing the same three prompting techniques used in Stage 1, we developed the fourth prompting technique specifically for Stage 2. The fourth prompting technique uses the Stage-1 predicted segmentation mask (generated with 0.0 threshold) of the first and every 50^*th*^ slice as the input prompt. For each of the four prompting techniques, we tested five logit thresholds: [−1, −0.5, 0, 0.5, 1]. The results are tabulated in Supplementary Tables S4, S5, S6, and S7. It shows that the newly introduced fourth prompting technique, with a logit threshold of −0.5, performs best and is therefore selected as the defaults for ETSAM Stage 2. The results also show that no logit threshold works best for all the tomograms. Therefore, we also provide users with an option to override the default logit threshold of Stage 2 during prediction, allowing them to achieve the preferred precision-recall trade-off on a per-tomogram basis.

Finally, for our last ablation study, we compared the results of ETSAM with only one stage and ETSAM with two stages on the test dataset, which are reported in Supplementary Tables S2 and S1, respectively. The two-stage ETSAM outperforms the one-stage ETSAM in terms of all metrics. While the quantitative improvement in evaluation metrics appears small, the three visualized examples in Supplementary Figure S3 show that the two-stage ETSAM substantially reduces small, scattered noise and visually improves the predicted membrane segmentation. Although adjusting logit thresholds can improve precision, it comes at the expense of lower recall scores. In contrast, ETSAM Stage 2 can improve Stage 1 predictions in terms of both recall and precision while also yielding notably less noisy results. This highlights the benefit of the two-stage ETSAM pipeline introduced in this study.

### Limitations.

ETSAM’s superior sensitivity effectively predicts most membrane regions, but it can also incorrectly treat some membrane-like noise artifacts as membranes in experimental tomograms. These noise artifacts can occur during cryo-ET sample preparation, imaging, or tomogram reconstruction. In the case of cryo-electron tomography of Schizosaccharomyces pombe 972h- cell (CDP Dataset - 10001, Run - 251), as shown in Supplementary Figure S4 (A), ETSAM, Membrain-Seg, and TARDIS predict a membrane-like artifact (false-positive) that is not an actual cell membrane. As shown in Supplementary Table S1, ETSAM accurately identified the ground-truth regions with a notable recall of 0.7697 for this tomogram, but it achieved a low precision of 0.3737 due to the false-positive prediction of membrane-like noise artifacts.

Similarly, Supplementary Figure S4 (B) shows another example in which ETSAM, Membrain-Seg, and TARDIS predict border artifacts as membranes to varying extents in the tomogram of a Halobacterium salinarum cell (CDP Dataset - 10099, Run - 7042). These types of false-positive membrane predictions require manual inspection and cleaning. This is a factor that negatively affects the prediction precision, which may be addressed in the future by more advanced AI models and larger training datasets with high quality ground truth annotations. Technological and scientific advancements in cryo-ET sample preparation, imaging and tomogram reconstruction can also significantly reduce noise artifacts introduced in tomograms.

## Conclusion

Accurate identification of cell membranes in cryo-ET tomograms is essential for understanding cellular architecture and function, yet remains challenging due to low signal-to-noise ratios, reconstruction artifacts, and the substantial effort required for manual annotation in large 3D volumes. In this study, we introduced ETSAM, a two-stage SAM2-based model that delivers fully automated, state-of-the-art segmentation of cell membrane in cryo-ET tomograms. ET-SAM achieved the best overall performance across evaluation metrics and provided a superior balance between precision and recall compared with existing deep-learning approaches. Its strong recall facilitates the detection of biologically relevant membrane regions, while its good precision limits noise and false positives, and can be further enhanced with post-processing. Together, these capabilities position ETSAM as a robust and practical tool for advancing structural and cellular studies using cryo-ET.

## Materials and Methods

### Data Collection.

The experimental cryo-ET tomograms and corresponding cell membrane annotations used in this work were collected from the CDP database (2). While most cell membrane annotations in the CDP database are generated using AI tools in a fully automated manner, some annotations are marked as ground truth as they have been further manually cleaned and reviewed by human annotators. Due to the limited availability, we initially curated a test dataset comprising 15 experimental tomograms from 6 cell datasets for which ground-truth cell membrane annotations are available. At most, only 3 tomograms were selected per cell dataset to avoid a single large cell dataset dominating the test dataset. To create the training dataset, we manually reviewed the experimental cryo-ET tomograms in the CDP database (2) and selected 83 experimental tomograms from 25 cell datasets with properly annotated cell membranes.

In addition to the experimental tomograms curated from the CDP database, we used PolNet to generate 38 simulated tomograms containing ground-truth cell membrane annotations. Of the 38 simulated tomograms, 28 were included in the training dataset and 10 in the test dataset for evaluation. In total, the curated training and test datasets contain 111 and 25 tomograms, respectively.

Furthermore, we additionally collected 5 experimental tomograms of different cellular organisms from the CDP database, which were not present in the training or test dataset. Even though these tomograms do not have ground-truth membrane annotations in the database, they were used to visually validate whether ETSAM could handle tomograms of new cellular organisms unseen during training.

### Dataset Preparation.

To enhance the quality of the training dataset, we manually cleaned the cell membrane annotations of the experimental tomograms to remove noise and false-positive membrane segmentation, as illustrated in Supplementary Figure S2. Moreover, since the density distributions of the experimental tomograms vary, we normalize them to the range 0.0 to 1.0 using min-max normalization.

As SAM2 (1) is designed to take 2D RGB image frames from videos as input, ETSAM also treats a 3D tomogram as a video and slices it along the Z-axis to produce image-like frames. Since an image is composed of RGB channels, ETSAM repeats each 2D slice of tomogram data across three channels to form the RGB channels in an image. Similarly, the 3D cell membrane annotation masks are also converted into 2D slices. Finally, the 2D image-like tomogram and corresponding membrane annotation slices are stored in input-label pairs to train the ETSAM model. Although ETSAM converts 3D tomograms into 2D image-like slices, it is worth noting that the tomogram data is not limited to 0–255 integer values like in traditional RGB images. Instead, they are stored as floating-point values (0.0 to 1.0) in NumPy (.npy) format, thereby preserving the precision of the tomogram data.

### ETSAM Membrane Segmentation Pipeline.

The ETSAM pipeline consists of two SAM2-based (1) blocks to perform two-stage membrane segmentation, as shown in Figure 5A. While the original SAM2 model was designed to take RGB image formats (.jpg/.png) as input with an integer value range of 0 to 255, ETSAM customizes it to load tomogram slices in floating-point values in the range [0.0, 1.0]. The input tomograms are normalized to the range by the min-max normalization. The normalized tomogram slices are first fed into the ETSAM Stage 1 block, which generates the initial membrane prediction. The first-stage initial segmentation is in the form of logits, whose values can range from negative to positive, with higher values indicating a greater likelihood of a membrane being present. The predicted logits are then clipped between −5 and +5 and normalized using min-max scaling. These normalized first-stage predicted logits are then concatenated with the normalized tomogram slices and fed as input to the ETSAM Stage 2 block, which generates the final membrane prediction.

As shown in Figure 5B, each ETSAM stage block features a SAM2 comprising an image encoder, a prompt encoder, a mask decoder, and specialized memory components, such as memory encoder and memory attention. The image encoder serves as the foundational component for feature extraction, utilizing a Hierarchical transformer (Hiera) architecture that processes input frames at multiple resolutions to capture both local details and global context. Unlike traditional Vision Transformers (ViT), Hiera employs a multi-stage design with quadtree-like downsampling, allowing for efficient hierarchical feature maps that reduce computational overhead while preserving spatial hierarchies. For video inputs, the encoder processes each frame independently, generating per-frame embeddings that form the basis for subsequent prompting and decoding.

The prompt encoder is responsible for encoding the user-provided prompts into the model’s latent space. It handles various prompt types — points, bounding boxes, and masks — by embedding them into high-dimensional vectors. In the prompt encoder, points and box prompts are encoded using positional embeddings, and mask prompts are passed through convolutional layers and positional encoding to align with the image encoder’s features. The memory encoder is a lightweight convolutional network that compresses mask predictions from previous frames into compact representations using fixed-dimensional tokens that capture essential spatial and semantic information. These tokens are then stored in the memory bank. A memory bank is a bounded buffer that stores representations of a fixed number of recent frames, using a first-in-first-out (FIFO) strategy to manage the memory footprint. Finally, the mask decoder is a lightweight bidirectional transformer with cross-attention and self-attention layers that integrate the encoded prompts with the image and memory bank features to predict the segmentation mask and its associated confidence score.

Both the ETSAM’s Stage 1 and 2 were initialized with the SAM2.1 base model checkpoint as the initial weights and fine-tuned on the training dataset for 602 and 545 epochs, respectively. During training, ETSAM uses the default random ground-truth-based prompting configuration from SAM2. It generates points, box, and mask prompts sampled from the ground truth annotations with a probability of 0.5, 0.25, and 0.25, respectively.

During inference, the ETSAM stage 1 automatically uses a fixed grid of points mask (dotted grid pattern) at the first slice as the input prompt. As shown in Figure 5A, the ETSAM Stage 1 predicted logits are converted into a binary mask by applying a default threshold of 0.0, and the binary mask of the first slice and every 50^*th*^ slice are used as the input prompt for ETSAM Stage 2. The prompting process for the two stages is fully automated. Finally, the logits predicted by the second-stage ETSAM block are converted into a binary segmentation mask by applying a default logit threshold of *−*0.5. Users can adjust this threshold to balance the precision and recall of the final prediction.

ETSAM was trained on NVIDIA A100 with a batch size of 1. Each batch corresponds to 22 consecutive 2D slices from a single tomogram. Apart from the above modifications, both stages of ETSAM use the same default model parameters and training regime as the original SAM2.

### Evaluation Metrics.

In the context of binary segmentation of cell membranes in a tomogram, TP (True Positives) are the correctly predicted positive pixels/membrane regions, FP (False Positives) are the regions incorrectly predicted as membranes (noise/over-segmentation), FN (False Negatives) are the missed membrane regions and TN (True Negatives) are the background (non-membrane) regions predicted correctly.

**Precision** measures the accuracy of a model’s positive prediction and is computed as the ratio of true positives to the total true positives and false positives. It ranges from 0 to 1, with 1 representing the highest precision, characterized by no false positive predictions, and 0 representing the lowest precision, where none of the positive regions are predicted correctly. It is calculated by the following equation:

$$\text{Precision}=\frac{TP}{TP+FP}$$

**Recall** measures the completeness of the model’s positive predictions and is computed as the ratio of true positives to the sum of true positives and false negatives. It ranges from 0 to 1, with 1 representing the highest recall, where all positive regions are predicted correctly, and 0 representing the lowest recall, where none of the positive regions are predicted. It is calculated as follows:

$$\text{Recall}=\frac{TP}{TP+FN}$$

**Dice Score**, also known as **F1 score**, is the harmonic mean of precision and recall, providing a single metric that balances both the accuracy of the prediction and its completeness. It ranges from 0 (no overlap) to 1 (perfect overlap) and penalizes extreme values, i.e., if either precision or recall is low, the Dice score will suffer significantly. Given two sets, *A* (prediction) and *B* (ground truth), it is calculated as follows:

$$\text{Dice}=\frac{2|A\cap B|}{\left|A+B\right|}=\frac{2\cdot TP}{2\cdot TP+FP+FN}$$

**Intersection over Union (IoU)** is the ratio of the area of overlap between the predicted segmentation and the ground truth to the area of their union, measuring the extent to which the two distinct sets share the same space. It ranges from 0 (no overlap) to 1 (perfect overlap), similar to the Dice score, but is slightly more stringent and effectively penalizes both false positives and false negatives. Given two sets, *A* (prediction) and *B* (ground truth), it is calculated as follows:

$$\text{IoU}=\frac{\left|A\cap B\right|}{\left|A\cup B\right|}=\frac{\;TP}{TP+FP+FN}$$

All the above evaluation metrics were used to provide a comprehensive analysis of ETSAM’s ability to effectively segment cell membranes in cryo-ET tomograms.

## Supplementary Material

Supplement 1

## Figures and Tables

**Table 1.** The comparison of performance between ETSAM and two other methods on 15 Experimental and 10 Simulated Tomograms in the test dataset in terms of multiple evaluation metrics.

	Experimental Tomograms				Simulated Tomograms			
Method	Dice/F1	IoU	Precision	Recall	Dice/F1	IoU	Precision	Recall
ETSAM	**0.5427**	**0.3796**	0.5175	**0.6269**	**0.6083**	**0.4385**	**0.5717**	0.6612
Membrain-Seg	0.4598	0.3090	0.3961	0.6245	0.5727	0.4022	0.4642	**0.7556**
TARDIS	0.4991	0.3434	**0.5205**	0.5661	0.5024	0.3364	0.4379	0.6032

Bold denotes the best result, and underline denotes the second best result.

**Table 2.** The results of the post-processed ETSAM membrane segmentation on the 15 experimental tomograms in the test dataset

Metrics	Score
Dice/F1	0.5436
IoU	0.3804
Precision	0.5192
Recall	0.6266

## Data Availability

The source code of ETSAM is freely available in GitHub. ETSAM model weights are available in Zenodo. The annotations and simulated tomograms used in training and test datasets are available in Harvard Dataverse. The experimental tomograms can be fetched from the CryoET Data Portal. We have also provided helper scripts to download the entire dataset and preprocess it in the GitHub repository.

## Significance Statement

Cryogenic Electron Tomography (cryo-ET) is an important technique for visualizing cellular structures in their native state. Effectively segmenting cell membranes in cryo-ET tomograms is vital for understanding cellular organization and function. The low signal-to-noise ratio and domain-specific artifacts in 3D cryo-ET tomograms hinder the analysis of the cell structures. In this study, we introduce ETSAM—a two-stage, SAM2-based fine-tuned AI model—that achieves state-ofthe-art performance in segmenting cell membranes from cryo-ET tomograms, providing a valuable tool for cryo-ET data analysis.
