# Supplementary material for: ETSAM: Effectively Segmenting Cell Membranes in cryo-Electron Tomograms
Source: bioRxiv. 2026 Mar 2:2025.11.23.689996. Originally published 2025 Nov 26. Preprint. [Version 3] doi: 10.1101/2025.11.23.689996 (PMC12697732; doi:10.1101/2025.11.23.689996)
Supplement: Supplementary file 2 [file media-2.pdf]

Supplementary Table 1: Per-Tomogram Performance of two-stage ETSAM on experimental tomograms in the test set

CDP IDs		ETSAM				TARDIS				Membrain-Seg			
Dataset	Run	Dice/F1	IoU	Precision	Recall	Dice/F1	IoU	Precision	Recall	Dice/F1	IoU	Precision	Recall
10000	245	0.7261	0.5700	0.8780	0.6191	0.5706	0.3992	0.9418	0.4093	0.7512	0.6015	0.7360	0.7670
10000	247	0.9595	0.9222	0.9623	0.9568	0.8139	0.6862	0.8291	0.7992	0.7875	0.6494	0.6837	0.9283
10001	258	0.9519	0.9082	0.9645	0.9396	0.7496	0.5995	0.9275	0.6289	0.8225	0.6985	0.8344	0.8109
10010	11972	0.8982	0.8152	0.8856	0.9112	0.7049	0.5443	0.8439	0.6052	0.7730	0.6300	0.7783	0.7679
10010	12000	0.9041	0.8250	0.9014	0.9068	0.6343	0.4645	0.5171	0.8203	0.6128	0.4418	0.4749	0.8635
10438	15936	0.6680	0.5015	0.6293	0.7119	0.5069	0.3395	0.6818	0.4034	0.5263	0.3571	0.4347	0.6668
10436	15943	0.7671	0.6222	0.8208	0.7200	0.5028	0.3358	0.6014	0.4320	0.5214	0.3526	0.4194	0.6890
10104	7325	0.3809	0.2352	0.2643	0.6811	0.0162	0.0081	0.0120	0.0246	0.0078	0.0039	0.0040	0.1135
10118	7667	0.8383	0.7216	0.7667	0.9246	0.4056	0.2544	0.2626	0.8901	0.1503	0.0812	0.0827	0.8195
10032	676	0.7726	0.6294	0.9430	0.6543	0.6485	0.4798	0.5842	0.7286	0.4459	0.2869	0.2927	0.9357
**AVERAGE**		0.7867	0.6751	0.8016	0.8025	0.5553	0.4111	0.6201	0.5742	0.5399	0.4103	0.4741	0.7362

Supplementary Table 2: Per-Tomogram Performance of two-stage ETSAM on experimental tomograms in test set after post-processing

CDP IDs		ETSAM			
**Dataset**	**Run**	**Dice/F1**	**IoU**	**Precision**	**Recall**
10000	245	0.7240	0.5674	0.8788	0.6156
10000	247	0.9606	0.9242	0.9646	0.9567
10001	258	0.9536	0.9114	0.9684	0.9393
10010	11972	0.9002	0.8185	0.8895	0.9112
10010	12000	0.9049	0.8263	0.9058	0.9041
10438	15936	0.6687	0.5023	0.6308	0.7116
10436	15943	0.7670	0.6220	0.8236	0.7176
10104	7325	0.3951	0.2462	0.2784	0.6803
10118	7667	0.8416	0.7265	0.7725	0.9242
10032	676	0.7709	0.6272	0.9444	0.6513
**AVERAGE**		0.7887	0.6772	0.8057	0.8012

Supplementary Table 3: Benchmark of runtime, CPU and GPU memory compute usage of all three methods evaluated on experimental tomograms in the test set

CDP IDs		ETSAM			TARDIS			Membrain-Seg		
**Dataset**	**Run**	**Time (seconds)**	**GPU Memory (MB)**	**CPU Memory (MB)**	**Time (seconds)**	**GPU Memory (MB)**	**CPU Memory (MB)**	**Time (seconds)**	**GPU Memory (MB)**	**CPU Memory (MB)**
10000	245	72.93	1350	20582	451.08	7906	8131	98.95	5520	20985
10000	247	73.36	1350	21160	129.19	7906	8328	104.60	5520	21169
10001	258	68.34	1350	21130	123.01	7906	7841	94.73	5520	21212
10010	11972	52.13	1348	9853	67.05	7906	5174	69.08	4772	12654
10010	12000	32.29	1394	6662	134.81	7906	4267	13.11	4034	4109
10438	15936	79.68	1310	17135	170.90	7906	10636	119.29	5804	24476
10436	15943	28.69	1394	6046	44.49	7906	2073	24.36	4110	4995
10104	7325	115.72	1350	17033	418.09	7906	10801	135.72	5520	21228
10118	7667	111.69	1350	25151	1741.01	7906	25683	141.20	6200	26013
10032	676	73.29	1350	19788	555.74	7906	13680	101.64	5520	21191
**AVERAGE**		70.81	1355	16454	383.54	7906	9661	90.27	5252	17803
**STD. DEVIATION**		28.67	24.19	6636.66	509.13	0.00	6577.15	43.05	713.41	7791.07

Supplementary Table 4: ETSAM Stage 1 Performance across different automated prompt techniques on experimental tomograms in the test set

CDP IDs		Zero Mask				Grid mask at only first slice				Grid mask at every 50th slice			
**Dataset**	**Run**	**Dice/F1**	**IoU**	**Precision**	**Recall**	**Dice/F1**	**IoU**	**Precision**	**Recall**	**Dice/F1**	**IoU**	**Precision**	**Recall**
10000	245	0.4942	0.3282	0.8208	0.3536	0.7423	0.5902	0.8573	0.6545	0.6274	0.4570	0.8864	0.4855
10000	247	0.0000	0.0000	0.0000	0.0000	0.8821	0.7891	0.8383	0.9308	0.8857	0.7948	0.9391	0.8381
10001	258	0.8987	0.8161	0.9177	0.8805	0.9192	0.8505	0.9131	0.9254	0.8420	0.7271	0.9666	0.7459
10010	11972	0.8380	0.7212	0.9642	0.7410	0.8634	0.7597	0.9045	0.8260	0.7050	0.5444	0.9876	0.5482
10010	12000	0.8484	0.7367	0.9002	0.8022	0.8772	0.7813	0.8899	0.8649	0.8099	0.6806	0.8904	0.7428
10438	15936	0.5930	0.4215	0.6490	0.5459	0.6590	0.4914	0.5937	0.7403	0.4930	0.3271	0.7305	0.3721
10436	15943	0.0000	0.0000	0.0000	0.0000	0.7555	0.6071	0.8710	0.6671	0.5809	0.4094	0.9092	0.4268
10104	7325	0.2361	0.1338	0.2188	0.2563	0.1660	0.0905	0.0947	0.6706	0.4342	0.2773	0.3903	0.4892
10118	7667	0.8193	0.6939	0.8327	0.8063	0.5265	0.3573	0.3878	0.8193	0.6582	0.4905	0.6675	0.6491
10032	676	0.0000	0.0000	0.0000	0.0000	0.7504	0.6005	0.9437	0.6228	0.5425	0.3722	0.9445	0.3805
**AVERAGE**		0.4728	0.3851	0.5303	0.4386	0.7142	0.5918	0.7294	0.7722	0.6579	0.5080	0.8312	0.5678

Supplementary Table 5: ETSAM Stage 2 Threshold Ablation with Prompt Technique - Zero Mask | Evaluated on experimental tomograms in the test set

CDP IDs		> -2.0				> -1.0				> -0.5				> -0.25			
**Dataset**	**Run**	**Dice/F1**	**IoU**	**Precision**	**Recall**	**Dice/F1**	**IoU**	**Precision**	**Recall**	**Dice/F1**	**IoU**	**Precision**	**Recall**	**Dice/F1**	**IoU**	**Precision**	**Recall**
10000	245	0.7542	0.6054	0.7822	0.7282	0.7506	0.6007	0.8443	0.6756	0.7376	0.5843	0.8696	0.6404	0.7261	0.5700	0.8780	0.6191
10000	247	0.8992	0.8169	0.8288	0.9827	0.9421	0.8905	0.9090	0.9776	0.9574	0.9182	0.9474	0.9675	0.9595	0.9222	0.9623	0.9568
10001	258	0.8388	0.7223	0.7240	0.9968	0.9289	0.8672	0.8733	0.9920	0.9559	0.9156	0.9435	0.9687	0.9519	0.9082	0.9645	0.9396
10010	11972	0.6956	0.5333	0.5398	0.9780	0.8225	0.6985	0.7219	0.9557	0.8800	0.7858	0.8325	0.9333	0.8982	0.8152	0.8856	0.9112
10010	12000	0.8018	0.6692	0.6879	0.9608	0.8731	0.7748	0.8121	0.9440	0.9004	0.8189	0.8763	0.9259	0.9041	0.8250	0.9014	0.9068
10438	15936	0.5634	0.3922	0.4258	0.8326	0.6349	0.4651	0.5336	0.7836	0.6623	0.4951	0.5983	0.7416	0.6680	0.5015	0.6293	0.7119
10436	15943	0.6718	0.5058	0.5436	0.8791	0.7473	0.5966	0.6910	0.8137	0.7687	0.6243	0.7788	0.7588	0.7671	0.6222	0.8208	0.7200
10104	7325	0.2418	0.1375	0.1418	0.8202	0.3249	0.1940	0.2067	0.7591	0.3642	0.2227	0.2448	0.7108	0.3809	0.2352	0.2643	0.6811
10118	7667	0.7679	0.6232	0.6317	0.9790	0.8169	0.6904	0.7101	0.9613	0.8338	0.7150	0.7491	0.9402	0.8383	0.7216	0.7667	0.9246
10032	676	0.7951	0.6598	0.8147	0.7763	0.7909	0.6542	0.8882	0.7129	0.7805	0.6400	0.9250	0.6751	0.7726	0.6294	0.9430	0.6543
**AVERAGE**		0.7030	0.5666	0.6120	0.8934	0.7632	0.6432	0.7190	0.8576	0.7841	0.6720	0.7765	0.8262	0.7867	0.6751	0.8016	0.8025

CDP IDs		> 0.0				> 0.25				> 0.5				> 1.0			
**Dataset**	**Run**	**Dice/F1**	**IoU**	**Precision**	**Recall**	**Dice/F1**	**IoU**	**Precision**	**Recall**	**Dice/F1**	**IoU**	**Precision**	**Recall**	**Dice/F1**	**IoU**	**Precision**	**Recall**
10000	245	0.7116	0.5523	0.8839	0.5955	0.6943	0.5318	0.8876	0.5702	0.6752	0.5097	0.8899	0.5440	0.6317	0.4617	0.8909	0.4894
10000	247	0.9567	0.9170	0.9730	0.9410	0.9497	0.9043	0.9800	0.9213	0.9397	0.8863	0.9844	0.8990	0.9147	0.8427	0.9895	0.8504
10001	258	0.9369	0.8812	0.9757	0.9010	0.9155	0.8442	0.9817	0.8577	0.8911	0.8036	0.9854	0.8133	0.8371	0.7199	0.9896	0.7254
10010	11972	0.8986	0.8158	0.9258	0.8729	0.8801	0.7859	0.9502	0.8197	0.8500	0.7391	0.9641	0.7600	0.7744	0.6319	0.9779	0.6410
10010	12000	0.8970	0.8133	0.9169	0.8780	0.8816	0.7883	0.9250	0.8421	0.8619	0.7574	0.9297	0.8034	0.8164	0.6898	0.9346	0.7248
10438	15936	0.6668	0.5002	0.6577	0.6763	0.6592	0.4916	0.6836	0.6365	0.6459	0.4770	0.7074	0.5943	0.6061	0.4348	0.7509	0.5081
10436	15943	0.7521	0.6027	0.8560	0.6707	0.7233	0.5665	0.8828	0.6126	0.6843	0.5202	0.9032	0.5509	0.5889	0.4174	0.9340	0.4301
10104	7325	0.3949	0.2460	0.2841	0.6472	0.4052	0.2541	0.3034	0.6096	0.4108	0.2585	0.3218	0.5679	0.4090	0.2570	0.3570	0.4786
10118	7667	0.8393	0.7231	0.7825	0.9051	0.8368	0.7195	0.7962	0.8819	0.8307	0.7104	0.8078	0.8549	0.8079	0.6777	0.8256	0.7909
10032	676	0.7618	0.6153	0.9595	0.6317	0.7473	0.5966	0.9734	0.6065	0.7283	0.5726	0.9832	0.5783	0.6788	0.5138	0.9921	0.5159
**AVERAGE**		0.7816	0.6667	0.8215	0.7719	0.7693	0.6483	0.8364	0.7358	0.7518	0.6235	0.8477	0.6966	0.7065	0.5647	0.8642	0.6155

CDP IDs		> 2.0			
**Dataset**	**Run**	**Dice/F1**	**IoU**	**Precision**	**Recall**
10000	245	0.5293	0.3599	0.8856	0.3774
10000	247	0.8534	0.7442	0.9943	0.7474
10001	258	0.7147	0.5561	0.9935	0.5581
10010	11972	0.6029	0.4315	0.9880	0.4338
10010	12000	0.7156	0.5571	0.9400	0.5777
10438	15936	0.4830	0.3184	0.8188	0.3425
10436	15943	0.3685	0.2259	0.9714	0.2274
10104	7325	0.3405	0.2052	0.4121	0.2901
10118	7667	0.7275	0.5718	0.8452	0.6386
10032	676	0.5587	0.3876	0.9975	0.3880
**AVERAGE**		0.5894	0.4358	0.8846	0.4581

Supplementary Table 6: ETSAM Stage 2 Threshold Ablation with Prompt Technique - Grid mask at every 50th slice | Evaluated on experimental tomograms in the test set

CDP IDs		> -2.0				> -1.0				> -0.5				> -0.25			
**Dataset**	**Run**	**Dice/F1**	**IoU**	**Precision**	**Recall**	**Dice/F1**	**IoU**	**Precision**	**Recall**	**Dice/F1**	**IoU**	**Precision**	**Recall**	**Dice/F1**	**IoU**	**Precision**	**Recall**
10000	245	0.6386	0.4690	0.8362	0.5165	0.5672	0.3959	0.8816	0.4181	0.5183	0.3498	0.8892	0.3657	0.4914	0.3257	0.8892	0.3395
10000	247	0.1835	0.1010	0.6207	0.1076	0.1719	0.0940	0.7121	0.0978	0.1627	0.0886	0.7479	0.0913	0.1572	0.0853	0.7632	0.0877
10001	258	0.8201	0.6950	0.7871	0.8558	0.8482	0.7364	0.9416	0.7717	0.7983	0.6642	0.9696	0.6784	0.7630	0.6169	0.9748	0.6268
10010	11972	0.7896	0.6523	0.6989	0.9073	0.8511	0.7407	0.9054	0.8029	0.8052	0.6739	0.9640	0.6913	0.7627	0.6164	0.9758	0.6260
10010	12000	0.8039	0.6721	0.7689	0.8423	0.8264	0.7042	0.8959	0.7669	0.8006	0.6675	0.9342	0.7004	0.7766	0.6348	0.9436	0.6599
10438	15936	0.4960	0.3298	0.5747	0.4362	0.4719	0.3089	0.6863	0.3596	0.4346	0.2776	0.7303	0.3093	0.4112	0.2588	0.7492	0.2833
10436	15943	0.6947	0.5323	0.6654	0.7268	0.6744	0.5087	0.8348	0.5657	0.6022	0.4308	0.8924	0.4544	0.5536	0.3827	0.9119	0.3974
10104	7325	0.3392	0.2043	0.2208	0.7319	0.4293	0.2733	0.3257	0.6298	0.4482	0.2888	0.3764	0.5538	0.4468	0.2877	0.3982	0.5091
10118	7667	0.7812	0.6409	0.6971	0.8884	0.8074	0.6770	0.7917	0.8237	0.7988	0.6650	0.8315	0.7686	0.7879	0.6501	0.8480	0.7358
10032	676	0.6805	0.5157	0.8661	0.5604	0.6075	0.4363	0.9538	0.4457	0.5440	0.3736	0.9749	0.3773	0.5078	0.3403	0.9803	0.3426
**AVERAGE**		0.6227	0.4812	0.6736	0.6573	0.6255	0.4875	0.7929	0.5682	0.5913	0.4480	0.8310	0.4991	0.5658	0.4199	0.8434	0.4608

CDP IDs		> 0.0				> 0.25				> 0.5				> 1.0			
**Dataset**	**Run**	**Dice/F1**	**IoU**	**Precision**	**Recall**	**Dice/F1**	**IoU**	**Precision**	**Recall**	**Dice/F1**	**IoU**	**Precision**	**Recall**	**Dice/F1**	**IoU**	**Precision**	**Recall**
10000	245	0.4634	0.3016	0.8869	0.3136	0.4352	0.2782	0.8836	0.2887	0.4066	0.2552	0.8787	0.2645	0.3495	0.2118	0.8655	0.2190
10000	247	0.1514	0.0819	0.7775	0.0839	0.1452	0.0783	0.7910	0.0800	0.1390	0.0747	0.8045	0.0760	0.1250	0.0667	0.8281	0.0676
10001	258	0.7246	0.5682	0.9782	0.5755	0.6841	0.5199	0.9804	0.5254	0.6424	0.4732	0.9819	0.4773	0.5572	0.3862	0.9834	0.3887
10010	11972	0.7142	0.5555	0.9813	0.5615	0.6631	0.4960	0.9840	0.5000	0.6113	0.4402	0.9857	0.4431	0.5096	0.3420	0.9876	0.3434
10010	12000	0.7483	0.5978	0.9484	0.6179	0.7180	0.5600	0.9515	0.5765	0.6862	0.5223	0.9539	0.5358	0.6219	0.4513	0.9582	0.4604
10438	15936	0.3859	0.2391	0.7663	0.2579	0.3592	0.2189	0.7812	0.2332	0.3318	0.1989	0.7942	0.2097	0.2771	0.1608	0.8154	0.1669
10436	15943	0.5015	0.3346	0.9272	0.3437	0.4476	0.2883	0.9393	0.2938	0.3942	0.2455	0.9494	0.2487	0.2928	0.1715	0.9647	0.1726
10104	7325	0.4385	0.2808	0.4174	0.4619	0.4239	0.2689	0.4339	0.4143	0.4032	0.2525	0.4478	0.3668	0.3489	0.2113	0.4665	0.2786
10118	7667	0.7723	0.6290	0.8616	0.6997	0.7527	0.6035	0.8727	0.6617	0.7289	0.5735	0.8812	0.6216	0.6727	0.5069	0.8917	0.5401
10032	676	0.4696	0.3069	0.9834	0.3085	0.4307	0.2744	0.9852	0.2756	0.3918	0.2436	0.9863	0.2444	0.3182	0.1892	0.9875	0.1896
**AVERAGE**		0.5370	0.3895	0.8528	0.4224	0.5060	0.3586	0.8603	0.3849	0.4735	0.3280	0.8664	0.3488	0.4073	0.2698	0.8749	0.2827

CDP IDs		> 2.0			
**Dataset**	**Run**	**Dice/F1**	**IoU**	**Precision**	**Recall**
10000	245	0.2413	0.1372	0.8232	0.1413
10000	247	0.0950	0.0499	0.8665	0.0503
10001	258	0.3921	0.2438	0.9836	0.2448
10010	11972	0.3253	0.1942	0.9880	0.1947
10010	12000	0.4943	0.3283	0.9658	0.3322
10438	15936	0.1788	0.0982	0.8426	0.1000
10436	15943	0.1354	0.0726	0.9835	0.0727
10104	7325	0.2248	0.1266	0.4804	0.1467
10118	7667	0.5345	0.3647	0.8959	0.3809
10032	676	0.2009	0.1116	0.9888	0.1118
**AVERAGE**		0.2822	0.1727	0.8818	0.1775

Supplementary Table 7: ETSAM Stage 2 Threshold Ablation with Prompt Technique - Grid mask at only first slice | Evaluated on experimental tomograms in the test set

CDP IDs		> -2.0				> -1.0				> -0.5				> -0.25			
**Dataset**	**Run**	**Dice/F1**	**IoU**	**Precision**	**Recall**	**Dice/F1**	**IoU**	**Precision**	**Recall**	**Dice/F1**	**IoU**	**Precision**	**Recall**	**Dice/F1**	**IoU**	**Precision**	**Recall**
10000	245	0.7543	0.6056	0.7622	0.7466	0.7555	0.6070	0.8298	0.6933	0.7476	0.5969	0.8612	0.6604	0.7387	0.5857	0.8730	0.6402
10000	247	0.8899	0.8017	0.8137	0.9818	0.9332	0.8747	0.8942	0.9756	0.9509	0.9063	0.9351	0.9672	0.9559	0.9155	0.9531	0.9587
10001	258	0.8267	0.7046	0.7058	0.9976	0.9200	0.8518	0.8560	0.9942	0.9572	0.9179	0.9352	0.9802	0.9581	0.9196	0.9609	0.9554
10010	11972	0.7150	0.5564	0.5634	0.9781	0.8317	0.7119	0.7354	0.9571	0.8867	0.7965	0.8412	0.9375	0.9058	0.8278	0.8933	0.9186
10010	12000	0.7956	0.6606	0.6781	0.9623	0.8699	0.7698	0.8057	0.9453	0.9003	0.8187	0.8736	0.9287	0.9052	0.8269	0.9003	0.9102
10438	15936	0.5477	0.3771	0.3963	0.8864	0.6266	0.4562	0.4991	0.8416	0.6633	0.4962	0.5633	0.8063	0.6758	0.5104	0.5957	0.7810
10436	15943	0.6692	0.5029	0.5389	0.8826	0.7442	0.5926	0.6846	0.8152	0.7666	0.6216	0.7721	0.7612	0.7672	0.6223	0.8154	0.7243
10104	7325	0.1589	0.0863	0.0880	0.8176	0.1939	0.1073	0.1110	0.7657	0.2092	0.1168	0.1223	0.7240	0.2153	0.1207	0.1274	0.6968
10118	7667	0.7538	0.6048	0.6117	0.9817	0.8072	0.6767	0.6929	0.9667	0.8282	0.7068	0.7347	0.9490	0.8353	0.7171	0.7544	0.9356
10032	676	0.7936	0.6578	0.8032	0.7841	0.7902	0.6532	0.8805	0.7168	0.7796	0.6389	0.9195	0.6767	0.7714	0.6279	0.9383	0.6549
**AVERAGE**		0.6905	0.5558	0.5961	0.9019	0.7472	0.6301	0.6989	0.8672	0.7690	0.6617	0.7558	0.8391	0.7729	0.6674	0.7812	0.8176

CDP IDs		> 0.0				> 0.25				> 0.5				> 1.0			
**Dataset**	**Run**	**Dice/F1**	**IoU**	**Precision**	**Recall**	**Dice/F1**	**IoU**	**Precision**	**Recall**	**Dice/F1**	**IoU**	**Precision**	**Recall**	**Dice/F1**	**IoU**	**Precision**	**Recall**
10000	245	0.7258	0.5696	0.8815	0.6169	0.7095	0.5498	0.8871	0.5911	0.6908	0.5276	0.8906	0.5642	0.6479	0.4791	0.8940	0.5080
10000	247	0.9564	0.9165	0.9672	0.9459	0.9520	0.9085	0.9772	0.9281	0.9435	0.8930	0.9836	0.9065	0.9189	0.8500	0.9900	0.8573
10001	258	0.9447	0.8952	0.9736	0.9174	0.9237	0.8583	0.9797	0.8738	0.8994	0.8172	0.9832	0.8288	0.8463	0.7335	0.9869	0.7407
10010	11972	0.9074	0.8305	0.9325	0.8836	0.8897	0.8013	0.9550	0.8327	0.8606	0.7554	0.9669	0.7754	0.7892	0.6518	0.9779	0.6615
10010	12000	0.8979	0.8146	0.9163	0.8801	0.8819	0.7887	0.9242	0.8432	0.8614	0.7565	0.9284	0.8034	0.8150	0.6878	0.9327	0.7238
10438	15936	0.6817	0.5171	0.6255	0.7489	0.6802	0.5153	0.6523	0.7105	0.6723	0.5063	0.6764	0.6682	0.6419	0.4727	0.7200	0.5791
10436	15943	0.7551	0.6066	0.8529	0.6774	0.7287	0.5731	0.8805	0.6215	0.6918	0.5288	0.9010	0.5615	0.6011	0.4297	0.9309	0.4439
10104	7325	0.2202	0.1237	0.1319	0.6649	0.2233	0.1257	0.1358	0.6277	0.2250	0.1268	0.1392	0.5865	0.2252	0.1269	0.1455	0.4975
10118	7667	0.8394	0.7233	0.7727	0.9187	0.8401	0.7243	0.7892	0.8981	0.8375	0.7204	0.8040	0.8739	0.8211	0.6965	0.8276	0.8147
10032	676	0.7603	0.6133	0.9561	0.6311	0.7455	0.5943	0.9711	0.6050	0.7258	0.5696	0.9818	0.5757	0.6746	0.5090	0.9918	0.5111
**AVERAGE**		0.7689	0.6610	0.8010	0.7885	0.7575	0.6439	0.8152	0.7532	0.7408	0.6202	0.8255	0.7144	0.6981	0.5637	0.8397	0.6338

CDP IDs		> 2.0			
**Dataset**	**Run**	**Dice/F1**	**IoU**	**Precision**	**Recall**
10000	245	0.5457	0.3752	0.8914	0.3932
10000	247	0.8559	0.7481	0.9949	0.7510
10001	258	0.7281	0.5724	0.9902	0.5757
10010	11972	0.6317	0.4616	0.9852	0.4648
10010	12000	0.7148	0.5562	0.9373	0.5777
10438	15936	0.5354	0.3655	0.7899	0.4049
10436	15943	0.3929	0.2445	0.9687	0.2464
10104	7325	0.2106	0.1177	0.1589	0.3122
10118	7667	0.7511	0.6014	0.8562	0.6689
10032	676	0.5520	0.3812	0.9981	0.3815
**AVERAGE**		0.5918	0.4424	0.8571	0.4776

Supplementary Table 8: ETSAM Stage 2 Threshold Ablation with Prompt Technique - Stage 1 mask at every 50th slice | Evaluated on experimental tomograms in the test set

CDP IDs		> -2.0				> -1.0				> -0.5				> -0.25			
**Dataset**	**Run**	**Dice/F1**	**IoU**	**Precision**	**Recall**	**Dice/F1**	**IoU**	**Precision**	**Recall**	**Dice/F1**	**IoU**	**Precision**	**Recall**	**Dice/F1**	**IoU**	**Precision**	**Recall**
10000	245	0.7743	0.6318	0.8133	0.7390	0.7701	0.6261	0.8711	0.6900	0.7629	0.6167	0.9004	0.6619	0.7486	0.5982	0.9038	0.6388
10000	247	0.9068	0.8295	0.8435	0.9803	0.9481	0.9012	0.9221	0.9755	0.9608	0.9245	0.9580	0.9635	0.9517	0.9078	0.9616	0.9420
10001	258	0.8564	0.7488	0.7498	0.9983	0.9398	0.8864	0.8883	0.9975	0.9812	0.9631	0.9688	0.9939	0.9639	0.9304	0.9744	0.9537
10010	11972	0.8087	0.6788	0.6936	0.9696	0.9030	0.8231	0.8657	0.9436	0.9432	0.8925	0.9657	0.9217	0.9159	0.8448	0.9717	0.8661
10010	12000	0.8322	0.7126	0.7369	0.9559	0.8955	0.8108	0.8554	0.9396	0.9225	0.8562	0.9191	0.9259	0.9066	0.8292	0.9238	0.8900
10438	15936	0.5924	0.4209	0.4518	0.8602	0.6562	0.4883	0.5480	0.8177	0.6853	0.5213	0.6056	0.7891	0.6829	0.5185	0.6229	0.7558
10436	15943	0.7309	0.5759	0.6525	0.8307	0.7735	0.6307	0.7898	0.7579	0.7782	0.6369	0.8693	0.7043	0.7504	0.6006	0.8837	0.6521
10104	7325	0.1985	0.1102	0.1131	0.8140	0.2414	0.1373	0.1435	0.7589	0.2571	0.1475	0.1569	0.7105	0.2616	0.1505	0.1620	0.6784
10118	7667	0.5564	0.3854	0.3900	0.9700	0.6033	0.4320	0.4451	0.9362	0.6175	0.4466	0.4696	0.9013	0.6211	0.4505	0.4804	0.8786
10032	676	0.7994	0.6658	0.8548	0.7507	0.7868	0.6486	0.9232	0.6856	0.7729	0.6298	0.9568	0.6483	0.7637	0.6177	0.9736	0.6282
**AVERAGE**		0.7056	0.5760	0.6299	0.8869	0.7518	0.6385	0.7252	0.8503	0.7682	0.6635	0.7770	0.8220	0.7566	0.6448	0.7858	0.7884

CDP IDs		> 0.0				> 0.25				> 0.5				> 1.0			
**Dataset**	**Run**	**Dice/F1**	**IoU**	**Precision**	**Recall**	**Dice/F1**	**IoU**	**Precision**	**Recall**	**Dice/F1**	**IoU**	**Precision**	**Recall**	**Dice/F1**	**IoU**	**Precision**	**Recall**
10000	245	0.7322	0.5776	0.9055	0.6147	0.7149	0.5563	0.9068	0.5901	0.6967	0.5346	0.9076	0.5653	0.6576	0.4899	0.9083	0.5154
10000	247	0.9404	0.8874	0.9632	0.9186	0.9279	0.8655	0.9641	0.8944	0.9147	0.8428	0.9645	0.8698	0.8860	0.7953	0.9648	0.8191
10001	258	0.9431	0.8923	0.9770	0.9114	0.9211	0.8538	0.9793	0.8695	0.8983	0.8154	0.9813	0.8282	0.8498	0.7388	0.9844	0.7476
10010	11972	0.8848	0.7934	0.9744	0.8103	0.8528	0.7433	0.9765	0.7569	0.8197	0.6945	0.9781	0.7055	0.7523	0.6030	0.9802	0.6104
10010	12000	0.8880	0.7986	0.9261	0.8530	0.8685	0.7675	0.9279	0.8162	0.8482	0.7365	0.9295	0.7800	0.8060	0.6750	0.9323	0.7098
10438	15936	0.6775	0.5123	0.6391	0.7207	0.6699	0.5037	0.6556	0.6850	0.6602	0.4927	0.6721	0.6487	0.6333	0.4634	0.7053	0.5747
10436	15943	0.7183	0.5604	0.8967	0.5990	0.6825	0.5180	0.9095	0.5462	0.6436	0.4744	0.9216	0.4944	0.5577	0.3867	0.9424	0.3960
10104	7325	0.2640	0.1521	0.1661	0.6428	0.2644	0.1523	0.1692	0.6042	0.2626	0.1512	0.1713	0.5629	0.2545	0.1458	0.1735	0.4775
10118	7667	0.6223	0.4517	0.4901	0.8522	0.6212	0.4505	0.4990	0.8227	0.6178	0.4470	0.5071	0.7904	0.6048	0.4335	0.5216	0.7196
10032	676	0.7521	0.6027	0.9889	0.6068	0.7298	0.5745	0.9920	0.5772	0.7053	0.5448	0.9933	0.5468	0.6538	0.4857	0.9956	0.4867
**AVERAGE**		0.7423	0.6229	0.7927	0.7530	0.7253	0.5985	0.7980	0.7162	0.7067	0.5734	0.8026	0.6792	0.6656	0.5217	0.8108	0.6057

CDP IDs		> 2.0			
**Dataset**	**Run**	**Dice/F1**	**IoU**	**Precision**	**Recall**
10000	245	0.5688	0.3974	0.9055	0.4146
10000	247	0.8213	0.6968	0.9646	0.7150
10001	258	0.7430	0.5911	0.9877	0.5955
10010	11972	0.6123	0.4412	0.9817	0.4449
10010	12000	0.7151	0.5566	0.9371	0.5781
10438	15936	0.5480	0.3774	0.7663	0.4265
10436	15943	0.3745	0.2304	0.9718	0.2319
10104	7325	0.2185	0.1226	0.1689	0.3094
10118	7667	0.5569	0.3859	0.5478	0.5664
10032	676	0.5389	0.3689	0.9987	0.3691
**AVERAGE**		0.5697	0.4168	0.8230	0.4651

Supplementary Table 9: Per-Tomogram Performance of ETSAM (single stage) on experimental tomograms in the test set

CDP IDs		ETSAM			
**Dataset**	**Run**	**Dice/F1**	**IoU**	**Precision**	**Recall**
10000	245	0.7423	0.5902	0.8573	0.6545
10000	247	0.8821	0.7891	0.8383	0.9308
10001	258	0.9192	0.8505	0.9131	0.9254
10010	11972	0.8634	0.7597	0.9045	0.8260
10010	12000	0.8772	0.7813	0.8899	0.8649
10438	15936	0.6590	0.4914	0.5937	0.7403
10436	15943	0.7555	0.6071	0.8710	0.6671
10104	7325	0.1660	0.0905	0.0947	0.6706
10118	7667	0.5265	0.3573	0.3878	0.8193
10032	676	0.7504	0.6005	0.9437	0.6228
**AVERAGE**		0.7142	0.5918	0.7294	0.7722
