# Supplementary material for: ETSAM: Effectively Segmenting Cell Membranes in cryo-Electron Tomograms
Source: bioRxiv. 2025 Nov 26:2025.11.23.689996. Preprint. [Version 1] doi: 10.1101/2025.11.23.689996 (PMC12697732; doi:10.1101/2025.11.23.689996)
Supplement: Supplementary file 1 [file media-1.pdf]

Supporting Information for

ETSAM: Effectively Segmenting Membranes in cryo-Electron Tomograms

Joel Selvaraj and Jianlin Cheng

Corresponding Author: Jianlin Cheng.
E-mail: chengji@missouri.edu

Table S1. Per-Tomogram Performance of two-stage ETSAM on experimental tomograms

	ETSAM				Membrain-Seg				TARDIS			
CDP Run ID	Dice	IoU	Precision	Recall	Dice	IoU	Precision	Recall	Dice	IoU	Precision	Recall
245	0.6482	0.4796	0.7315	0.5820	0.6425	0.4733	0.6533	0.6321	0.6086	0.4374	0.8267	0.4815
246	0.5661	0.3948	0.6619	0.4945	0.5319	0.3623	0.5241	0.5399	0.5137	0.3456	0.7455	0.3919
247	0.6987	0.5369	0.8327	0.6018	0.6547	0.4866	0.7219	0.5989	0.7296	0.5743	0.8344	0.6482
251	0.5312	0.3616	0.4038	0.7758	0.3103	0.1837	0.1965	0.7371	0.5340	0.3642	0.3916	0.8391
254	0.6223	0.4517	0.6467	0.5996	0.3762	0.2316	0.2921	0.5283	0.6436	0.4745	0.6549	0.6327
258	0.6500	0.4815	0.6718	0.6295	0.6414	0.4721	0.7257	0.5746	0.6298	0.4597	0.7567	0.5394
11972	0.6233	0.4528	0.4884	0.8613	0.6261	0.4557	0.4848	0.8836	0.6739	0.5082	0.5494	0.8713
11988	0.5759	0.4044	0.4143	0.9442	0.5779	0.4063	0.4084	0.9877	0.4545	0.2941	0.2959	0.9791
12000	0.6396	0.4702	0.5000	0.8873	0.4398	0.2819	0.2885	0.9249	0.4263	0.2709	0.2787	0.9064
15943	0.4184	0.2646	0.4785	0.3717	0.3698	0.2268	0.3250	0.4289	0.3487	0.2112	0.3633	0.3352
16039	0.4532	0.2930	0.4914	0.4205	0.4411	0.2830	0.3693	0.5477	0.4002	0.2501	0.4993	0.3339
16114	0.3242	0.1935	0.3367	0.3126	0.3145	0.1866	0.2381	0.4629	0.3032	0.1787	0.4534	0.2278
15936	0.4811	0.3167	0.3666	0.6997	0.4230	0.2682	0.3157	0.6407	0.5435	0.3731	0.5296	0.5581
15937	0.5239	0.3549	0.4574	0.6129	0.2664	0.1537	0.1978	0.4078	0.3789	0.2337	0.3753	0.3825
15939	0.3847	0.2381	0.2809	0.6102	0.2811	0.1635	0.2001	0.4721	0.2986	0.1755	0.2532	0.3639
Mean	0.5427	0.3796	0.5175	0.6269	0.4598	0.3090	0.3961	0.6245	0.4991	0.3434	0.5205	0.5661

Evaluated on 15 CryoET Data Portal (CDP) experimental tomograms from the test dataset.

Table S2. Per-Tomogram Performance of ETSAM (one stage) on experimental tomograms

CDP Run ID	Dice	IoU	Precision	Recall
245	0.6433	0.4742	0.7117	0.5870
246	0.5451	0.3747	0.6130	0.4907
247	0.6617	0.4944	0.7459	0.5945
251	0.5273	0.3580	0.3998	0.7740
254	0.6053	0.4340	0.6089	0.6016
258	0.6327	0.4628	0.6579	0.6095
11972	0.6261	0.4557	0.4988	0.8408
11988	0.5893	0.4177	0.4280	0.9453
12000	0.6449	0.4760	0.5096	0.8781
15943	0.4100	0.2579	0.4852	0.3550
16039	0.4364	0.2791	0.4612	0.4142
16114	0.3359	0.2019	0.3342	0.3377
15936	0.4796	0.3155	0.3712	0.6777
15937	0.5250	0.3559	0.4708	0.5934
15939	0.3828	0.2367	0.2881	0.5703
Mean	0.5364	0.3730	0.5056	0.6180

Evaluated on 15 CryoET Data Portal (CDP) experimental tomograms from the test dataset.

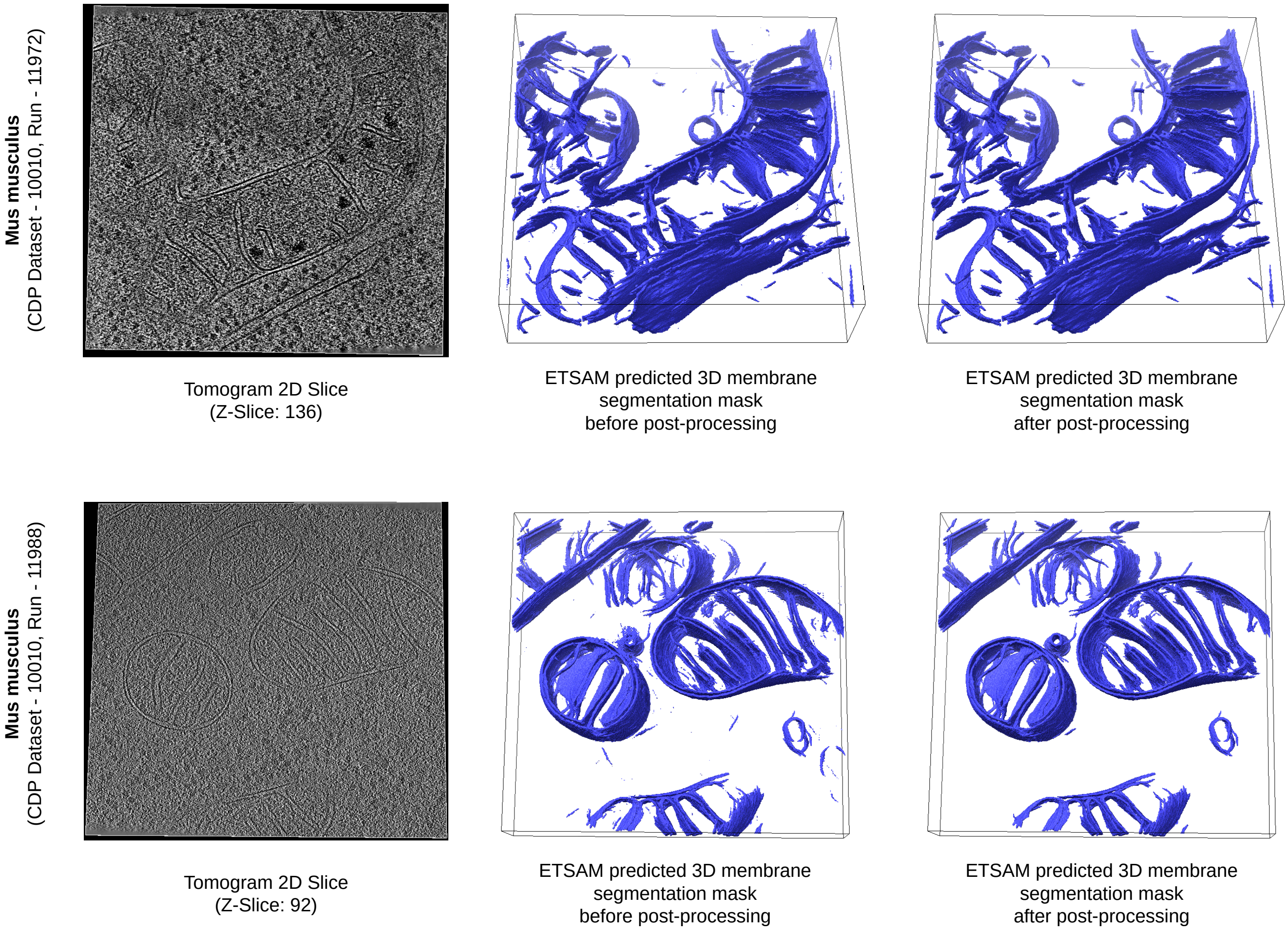

Fig. S1. Visual improvements of post-processing ETSAM Membrane Segmentations observed in cryo-electron tomograms of Mus musculus cell (CDP Dataset - 10010, Run - 11972 and 11988).

Table S3. ETSAM Stage 1 Performance across different automated prompt techniques

	Zero Mask				Grid mask at only first slice				Grid mask at every 50th slice			
CDP Run ID	Dice	IoU	Precision	Recall	Dice	IoU	Precision	Recall	Dice	IoU	Precision	Recall
245	0.4190	0.2650	0.6579	0.3074	0.6433	0.4742	0.7117	0.5870	0.5771	0.4056	0.7749	0.4597
246	0.4589	0.2978	0.6778	0.3469	0.5451	0.3747	0.6130	0.4907	0.4388	0.2811	0.7621	0.3081
247	0.0000	0.0000	0.0000	0.0000	0.6617	0.4944	0.7459	0.5945	0.6852	0.5212	0.8792	0.5614
251	0.5238	0.3549	0.4093	0.7274	0.5273	0.3580	0.3998	0.7740	0.5590	0.3879	0.4550	0.7246
254	0.4209	0.2665	0.6725	0.3063	0.6053	0.4340	0.6089	0.6016	0.5908	0.4193	0.7607	0.4830
258	0.6259	0.4555	0.6694	0.5877	0.6327	0.4628	0.6579	0.6095	0.6195	0.4487	0.7491	0.5281
11972	0.6603	0.4928	0.5630	0.7982	0.6261	0.4557	0.4988	0.8408	0.6640	0.4970	0.6561	0.6720
11988	0.6044	0.4331	0.4467	0.9346	0.5893	0.4177	0.4280	0.9453	0.6465	0.4777	0.5352	0.8165
12000	0.6633	0.4962	0.5436	0.8506	0.6449	0.4760	0.5096	0.8781	0.6473	0.4785	0.5455	0.7958
15943	0.0000	0.0000	0.0000	0.0000	0.4100	0.2579	0.4852	0.3550	0.3132	0.1857	0.5121	0.2256
16039	0.4145	0.2614	0.5076	0.3502	0.4364	0.2791	0.4612	0.4142	0.2821	0.1642	0.6022	0.1842
16114	0.3015	0.1775	0.3511	0.2641	0.3359	0.2019	0.3342	0.3377	0.2123	0.1187	0.5158	0.1336
15936	0.5361	0.3662	0.4869	0.5964	0.4796	0.3155	0.3712	0.6777	0.4694	0.3067	0.5489	0.4101
15937	0.5187	0.3502	0.5038	0.5346	0.5250	0.3559	0.4708	0.5934	0.4390	0.2812	0.5155	0.3823
15939	0.2775	0.1611	0.2664	0.2896	0.3828	0.2367	0.2881	0.5703	0.3468	0.2098	0.3687	0.3273
Mean	0.4283	0.2919	0.4504	0.4596	0.5364	0.3730	0.5056	0.6180	0.4994	0.3456	0.6121	0.4675

Table S4. ETSAM Stage 2 Threshold Ablation with Prompt Technique - Zero Mask

	> -1.0				> -0.5				> 0.0				> 0.5				> 1.0			
CDP Run ID	Dice	IoU	Precision	Recall	Dice	IoU	Precision	Recall	Dice	IoU	Precision	Recall	Dice	IoU	Precision	Recall	Dice	IoU	Precision	Recall
245	0.6445	0.4754	0.6939	0.6016	0.6332	0.4633	0.7134	0.5692	0.6176	0.4468	0.7320	0.5341	0.5973	0.4258	0.7494	0.4965	0.5707	0.3993	0.7647	0.4552
246	0.5704	0.3990	0.5870	0.5548	0.5637	0.3925	0.6388	0.5044	0.5445	0.3741	0.6879	0.4506	0.5122	0.3443	0.7326	0.3938	0.4668	0.3044	0.7710	0.3347
247	0.7311	0.5762	0.8387	0.6480	0.7202	0.5627	0.8506	0.6244	0.7074	0.5473	0.8621	0.5998	0.6927	0.5299	0.8730	0.5741	0.6761	0.5107	0.8837	0.5474
251	0.5131	0.3450	0.3842	0.7719	0.5254	0.3563	0.4035	0.7527	0.5359	0.3660	0.4228	0.7314	0.5442	0.3738	0.4420	0.7078	0.5502	0.3795	0.4612	0.6819
254	0.6162	0.4453	0.6328	0.6005	0.6149	0.4439	0.6668	0.5704	0.6088	0.4376	0.6998	0.5387	0.5977	0.4262	0.7320	0.5051	0.5815	0.4100	0.7629	0.4698
258	0.6515	0.4832	0.6396	0.6639	0.6476	0.4788	0.6689	0.6276	0.6383	0.4687	0.6973	0.5885	0.6231	0.4525	0.7244	0.5466	0.6012	0.4298	0.7491	0.5021
11972	0.5351	0.3653	0.3771	0.9207	0.5776	0.4060	0.4284	0.8861	0.6131	0.4421	0.4827	0.8401	0.6378	0.4683	0.5380	0.7833	0.6482	0.4795	0.5917	0.7167
11988	0.4964	0.3302	0.3344	0.9630	0.5478	0.3773	0.3845	0.9527	0.6013	0.4299	0.4429	0.9362	0.6529	0.4847	0.5098	0.9077	0.6954	0.5330	0.5845	0.8581
12000	0.5991	0.4277	0.4457	0.9139	0.6257	0.4553	0.4807	0.8958	0.6492	0.4806	0.5168	0.8730	0.6675	0.5010	0.5528	0.8424	0.6789	0.5139	0.5876	0.8039
15943	0.4478	0.2885	0.4223	0.4765	0.4374	0.2800	0.4531	0.4229	0.4149	0.2617	0.4839	0.3631	0.3791	0.2339	0.5142	0.3003	0.3330	0.1998	0.5443	0.2399
16039	0.5160	0.3477	0.4736	0.5667	0.4868	0.3217	0.5088	0.4666	0.4353	0.2782	0.5456	0.3622	0.3651	0.2233	0.5861	0.2651	0.2838	0.1654	0.6293	0.1832
16114	0.3519	0.2135	0.3107	0.4057	0.3280	0.1961	0.3272	0.3288	0.2912	0.1704	0.3417	0.2537	0.2433	0.1385	0.3525	0.1857	0.1924	0.1064	0.3613	0.1311
15936	0.4789	0.3148	0.3509	0.7538	0.5026	0.3356	0.3898	0.7071	0.5193	0.3507	0.4320	0.6507	0.5238	0.3548	0.4746	0.5844	0.5129	0.3449	0.5149	0.5109
15937	0.5146	0.3464	0.4205	0.6628	0.5211	0.3523	0.4451	0.6284	0.5238	0.3548	0.4719	0.5886	0.5211	0.3524	0.5007	0.5432	0.5113	0.3435	0.5305	0.4935
15939	0.3490	0.2114	0.2346	0.6811	0.3737	0.2298	0.2690	0.6120	0.3908	0.2428	0.3100	0.5286	0.3910	0.2430	0.3521	0.4395	0.3666	0.2244	0.3873	0.3480
Mean	0.5344	0.3713	0.4764	0.6790	0.5404	0.3768	0.5086	0.6366	0.5394	0.3768	0.5420	0.5893	0.5299	0.3702	0.5756	0.5384	0.5113	0.3563	0.6083	0.4851

Table S5. ETSAM Stage 2 Threshold Ablation with Prompt Technique - Grid mask at every 50th slice

	> -1.0				> -0.5				> 0.0				> 0.5				> 1.0			
CDP Run ID	Dice	IoU	Precision	Recall	Dice	IoU	Precision	Recall	Dice	IoU	Precision	Recall	Dice	IoU	Precision	Recall	Dice	IoU	Precision	Recall
245	0.4971	0.3308	0.7390	0.3745	0.4608	0.2994	0.7546	0.3317	0.4208	0.2665	0.7668	0.2900	0.3775	0.2326	0.7746	0.2495	0.3312	0.1985	0.7766	0.2105
246	0.4876	0.3224	0.6557	0.3881	0.4466	0.2875	0.7162	0.3245	0.3878	0.2405	0.7660	0.2596	0.3165	0.1880	0.8031	0.1971	0.2423	0.1378	0.8298	0.1418
247	0.1086	0.0574	0.5853	0.0598	0.1015	0.0535	0.6116	0.0554	0.0941	0.0494	0.6357	0.0508	0.0860	0.0449	0.6573	0.0460	0.0772	0.0402	0.6752	0.0410
251	0.5330	0.3633	0.4147	0.7458	0.5491	0.3785	0.4449	0.7171	0.5603	0.3892	0.4742	0.6846	0.5663	0.3950	0.5030	0.6479	0.5657	0.3944	0.5299	0.6068
254	0.5813	0.4097	0.6278	0.5412	0.5714	0.3999	0.6796	0.4929	0.5490	0.3784	0.7273	0.4409	0.5140	0.3459	0.7705	0.3856	0.4670	0.3046	0.8083	0.3283
258	0.5957	0.4242	0.6952	0.5211	0.5684	0.3971	0.7282	0.4662	0.5296	0.3602	0.7566	0.4074	0.4796	0.3154	0.7786	0.3465	0.4224	0.2678	0.7946	0.2877
11972	0.6051	0.4338	0.4875	0.7975	0.6238	0.4533	0.5476	0.7246	0.6201	0.4494	0.6039	0.6373	0.5927	0.4211	0.6539	0.5419	0.5457	0.3752	0.6985	0.4477
11988	0.4796	0.3154	0.4609	0.4998	0.4940	0.3280	0.5267	0.4651	0.4898	0.3243	0.5961	0.4157	0.4613	0.2998	0.6669	0.3525	0.4049	0.2538	0.7361	0.2792
12000	0.6049	0.4336	0.5026	0.7594	0.6160	0.4451	0.5408	0.7155	0.6172	0.4463	0.5771	0.6633	0.6079	0.4367	0.6108	0.6051	0.5901	0.4186	0.6430	0.5453
15943	0.3573	0.2175	0.4541	0.2946	0.3164	0.1879	0.4824	0.2354	0.2682	0.1549	0.5116	0.1818	0.2164	0.1213	0.5390	0.1354	0.1655	0.0902	0.5655	0.0970
16039	0.3665	0.2243	0.5295	0.2802	0.2829	0.1647	0.5624	0.1890	0.1933	0.1070	0.5927	0.1155	0.1189	0.0632	0.6144	0.0658	0.0701	0.0363	0.6210	0.0372
16114	0.2964	0.1740	0.4193	0.2292	0.2380	0.1351	0.4571	0.1609	0.1734	0.0949	0.4888	0.1054	0.1166	0.0619	0.5037	0.0659	0.0744	0.0386	0.4908	0.0402
15936	0.2668	0.1539	0.3238	0.2269	0.2534	0.1451	0.3517	0.1980	0.2311	0.1306	0.3748	0.1670	0.2029	0.1129	0.3920	0.1368	0.1719	0.0940	0.4028	0.1093
15937	0.4991	0.3325	0.5043	0.4940	0.4848	0.3200	0.5330	0.4446	0.4605	0.2992	0.5615	0.3903	0.4280	0.2723	0.5903	0.3357	0.3889	0.2414	0.6190	0.2836
15939	0.3840	0.2376	0.3050	0.5182	0.3816	0.2358	0.3443	0.4279	0.3614	0.2205	0.3852	0.3403	0.3242	0.1934	0.4265	0.2615	0.2706	0.1565	0.4602	0.1917
Mean	0.4442	0.2954	0.5136	0.4487	0.4259	0.2821	0.5521	0.3966	0.3971	0.2608	0.5879	0.3433	0.3606	0.2336	0.6190	0.2916	0.3192	0.2032	0.6434	0.2432

Table S6. ETSAM Stage 2 Threshold Ablation with Prompt Technique - Grid mask at only first slice

	> -1.0				> -0.5				> 0.0				> 0.5				> 1.0			
CDP Run ID	Dice	IoU	Precision	Recall	Dice	IoU	Precision	Recall	Dice	IoU	Precision	Recall	Dice	IoU	Precision	Recall	Dice	IoU	Precision	Recall
245	0.6494	0.4808	0.6835	0.6185	0.6395	0.4700	0.7046	0.5853	0.6250	0.4546	0.7248	0.5494	0.6055	0.4342	0.7439	0.5106	0.5804	0.4089	0.7614	0.4690
246	0.5759	0.4044	0.5980	0.5554	0.5676	0.3963	0.6453	0.5066	0.5481	0.3775	0.6899	0.4546	0.5171	0.3487	0.7299	0.4004	0.4753	0.3118	0.7646	0.3449
247	0.7365	0.5829	0.8334	0.6597	0.7259	0.5697	0.8465	0.6354	0.7136	0.5547	0.8591	0.6102	0.6990	0.5373	0.8713	0.5836	0.6822	0.5177	0.8828	0.5558
251	0.5137	0.3456	0.3760	0.8109	0.5288	0.3594	0.3967	0.7929	0.5418	0.3716	0.4172	0.7728	0.5527	0.3819	0.4374	0.7507	0.5614	0.3902	0.4575	0.7263
254	0.6033	0.4319	0.6007	0.6059	0.6065	0.4352	0.6421	0.5746	0.6043	0.4329	0.6836	0.5414	0.5957	0.4242	0.7238	0.5061	0.5810	0.4095	0.7620	0.4696
258	0.6555	0.4876	0.6373	0.6748	0.6530	0.4847	0.6681	0.6385	0.6449	0.4759	0.6976	0.5995	0.6305	0.4604	0.7252	0.5578	0.6096	0.4385	0.7499	0.5136
11972	0.5419	0.3717	0.3838	0.9219	0.5821	0.4105	0.4325	0.8899	0.6166	0.4458	0.4845	0.8478	0.6416	0.4724	0.5374	0.7961	0.6538	0.4857	0.5885	0.7355
11988	0.4929	0.3270	0.3314	0.9617	0.5420	0.3718	0.3790	0.9514	0.5925	0.4210	0.4335	0.9356	0.6416	0.4723	0.4954	0.9100	0.6838	0.5196	0.5642	0.8679
12000	0.5962	0.4247	0.4423	0.9145	0.6236	0.4531	0.4783	0.8959	0.6477	0.4790	0.5152	0.8721	0.6658	0.4991	0.5512	0.8407	0.6770	0.5117	0.5860	0.8015
15943	0.4447	0.2860	0.4172	0.4762	0.4338	0.2770	0.4466	0.4218	0.4113	0.2589	0.4759	0.3622	0.3775	0.2327	0.5050	0.3014	0.3347	0.2010	0.5338	0.2438
16039	0.4921	0.3264	0.4144	0.6058	0.4760	0.3123	0.4493	0.5061	0.4389	0.2812	0.4863	0.4000	0.3820	0.2361	0.5271	0.2995	0.3105	0.1838	0.5714	0.2132
16114	0.3555	0.2162	0.2957	0.4457	0.3391	0.2041	0.3135	0.3691	0.3104	0.1837	0.3307	0.2924	0.2693	0.1556	0.3451	0.2208	0.2211	0.1243	0.3571	0.1601
15936	0.4476	0.2884	0.3145	0.7759	0.4727	0.3095	0.3492	0.7313	0.4921	0.3264	0.3865	0.6773	0.5019	0.3350	0.4244	0.6141	0.4990	0.3325	0.4613	0.5435
15937	0.5159	0.3476	0.4203	0.6678	0.5233	0.3544	0.4457	0.6336	0.5269	0.3577	0.4736	0.5938	0.5249	0.3559	0.5033	0.5486	0.5159	0.3477	0.5336	0.4994
15939	0.3608	0.2201	0.2457	0.6793	0.3843	0.2378	0.2801	0.6121	0.3978	0.2483	0.3177	0.5319	0.3942	0.2455	0.3527	0.4467	0.3707	0.2275	0.3813	0.3607
Mean	0.5321	0.3694	0.4663	0.6916	0.5399	0.3764	0.4985	0.6497	0.5408	0.3779	0.5317	0.6027	0.5333	0.3727	0.5649	0.5525	0.5171	0.3607	0.5970	0.5003

Table S7. ETSAM Stage 2 Threshold Ablation with Prompt Technique - Stage 1 mask at every 50th slice

	> -1.0				> -0.5				> 0.0				> 0.5				> 1.0			
CDP Run ID	Dice	IoU	Precision	Recall	Dice	IoU	Precision	Recall	Dice	IoU	Precision	Recall	Dice	IoU	Precision	Recall	Dice	IoU	Precision	Recall
245	0.6588	0.4912	0.7136	0.6118	0.6482	0.4796	0.7315	0.5820	0.6342	0.4644	0.7487	0.5501	0.6162	0.4453	0.7649	0.5160	0.5941	0.4226	0.7801	0.4797
246	0.5783	0.4068	0.6274	0.5364	0.5661	0.3948	0.6619	0.4945	0.5466	0.3761	0.6940	0.4509	0.5204	0.3517	0.7238	0.4063	0.4870	0.3219	0.7506	0.3604
247	0.7108	0.5514	0.8232	0.6255	0.6987	0.5369	0.8327	0.6018	0.6849	0.5209	0.8420	0.5773	0.6694	0.5030	0.8509	0.5517	0.6519	0.4836	0.8597	0.5250
251	0.5185	0.3500	0.3847	0.7952	0.5312	0.3616	0.4038	0.7758	0.5421	0.3718	0.4231	0.7542	0.5511	0.3804	0.4424	0.7306	0.5580	0.3869	0.4618	0.7048
254	0.6206	0.4499	0.6128	0.6286	0.6223	0.4517	0.6467	0.5996	0.6195	0.4488	0.6803	0.5687	0.6117	0.4406	0.7129	0.5357	0.5987	0.4273	0.7444	0.5008
258	0.6539	0.4858	0.6458	0.6623	0.6500	0.4815	0.6718	0.6295	0.6418	0.4725	0.6974	0.5943	0.6285	0.4583	0.7217	0.5567	0.6097	0.4385	0.7440	0.5164
11972	0.5953	0.4238	0.4455	0.8972	0.6233	0.4528	0.4884	0.8613	0.6445	0.4755	0.5319	0.8176	0.6566	0.4888	0.5746	0.7661	0.6581	0.4904	0.6148	0.7078
11988	0.5337	0.3640	0.3704	0.9547	0.5759	0.4044	0.4143	0.9442	0.6181	0.4473	0.4632	0.9286	0.6580	0.4903	0.5173	0.9040	0.6920	0.5291	0.5762	0.8661
12000	0.6170	0.4461	0.4679	0.9055	0.6396	0.4702	0.5000	0.8873	0.6590	0.4914	0.5326	0.8641	0.6736	0.5078	0.5646	0.8346	0.6820	0.5175	0.5954	0.7982
15943	0.4354	0.2783	0.4542	0.4180	0.4184	0.2646	0.4785	0.3717	0.3922	0.2439	0.5023	0.3216	0.3565	0.2169	0.5250	0.2698	0.3136	0.1859	0.5465	0.2199
16039	0.4813	0.3169	0.4628	0.5013	0.4532	0.2930	0.4914	0.4205	0.4124	0.2597	0.5206	0.3414	0.3608	0.2201	0.5497	0.2685	0.3011	0.1772	0.5757	0.2038
16114	0.3442	0.2079	0.3222	0.3693	0.3242	0.1935	0.3367	0.3126	0.2966	0.1741	0.3492	0.2578	0.2627	0.1512	0.3589	0.2072	0.2251	0.1268	0.3652	0.1627
15936	0.4638	0.3019	0.3380	0.7386	0.4811	0.3167	0.3666	0.6996	0.4939	0.3279	0.3964	0.6549	0.5000	0.3334	0.4267	0.6037	0.4974	0.3310	0.4569	0.5457
15937	0.5200	0.3513	0.4376	0.6407	0.5239	0.3549	0.4574	0.6129	0.5247	0.3557	0.4785	0.5808	0.5214	0.3526	0.5009	0.5436	0.5130	0.3450	0.5252	0.5014
15939	0.3658	0.2238	0.2518	0.6679	0.3847	0.2381	0.2809	0.6102	0.3962	0.2471	0.3120	0.5428	0.3967	0.2474	0.3425	0.4712	0.3827	0.2366	0.3683	0.3983
Mean	0.5398	0.3766	0.4905	0.6635	0.5427	0.3796	0.5175	0.6269	0.5405	0.3785	0.5448	0.5870	0.5322	0.3725	0.5718	0.5444	0.5176	0.3614	0.5976	0.4994

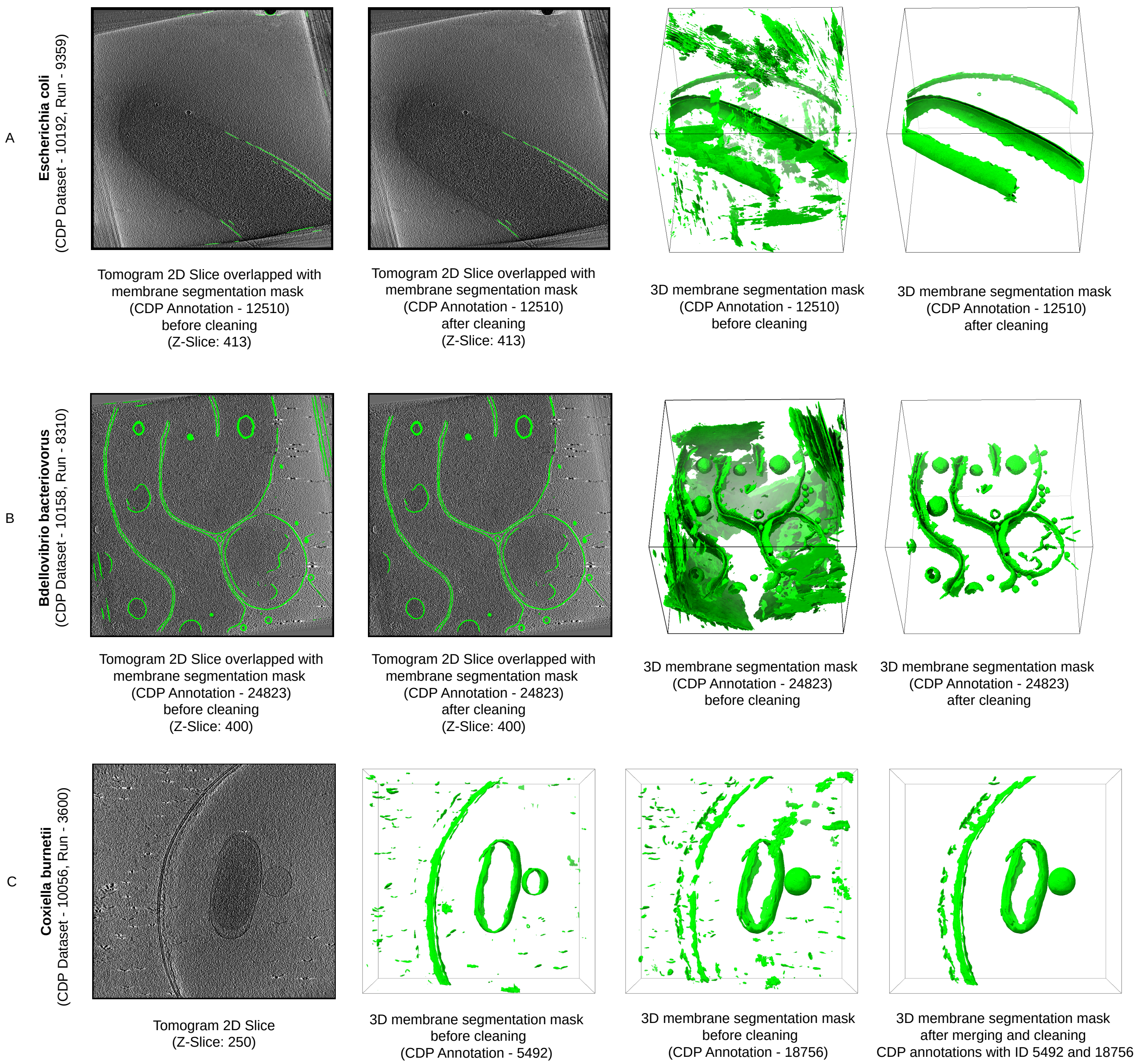

Fig. S2. Visualization of the improvements of manually processing membrane annotations of three experimental tomograms from our training dataset. (A) compares the membrane segmentation of the Escherichia coli cell (CDP Dataset - 10192, Run - 9359) before and after cleaning random noise. (B) compares the membrane segmentation of the Bdellovibrio bacteriovorus cell (CDP Dataset - 10158, Run - 8310) before and after cleaning the border artifacts. (C) shows an example of two different membrane segmentations (CDP Annotation - 5492 and 18756) for the same tomogram of a Coxiella burnetii cell (CDP Dataset - 10056, Run - 3600) being cleaned and combined to form the final segmentation used during ETSAM training.

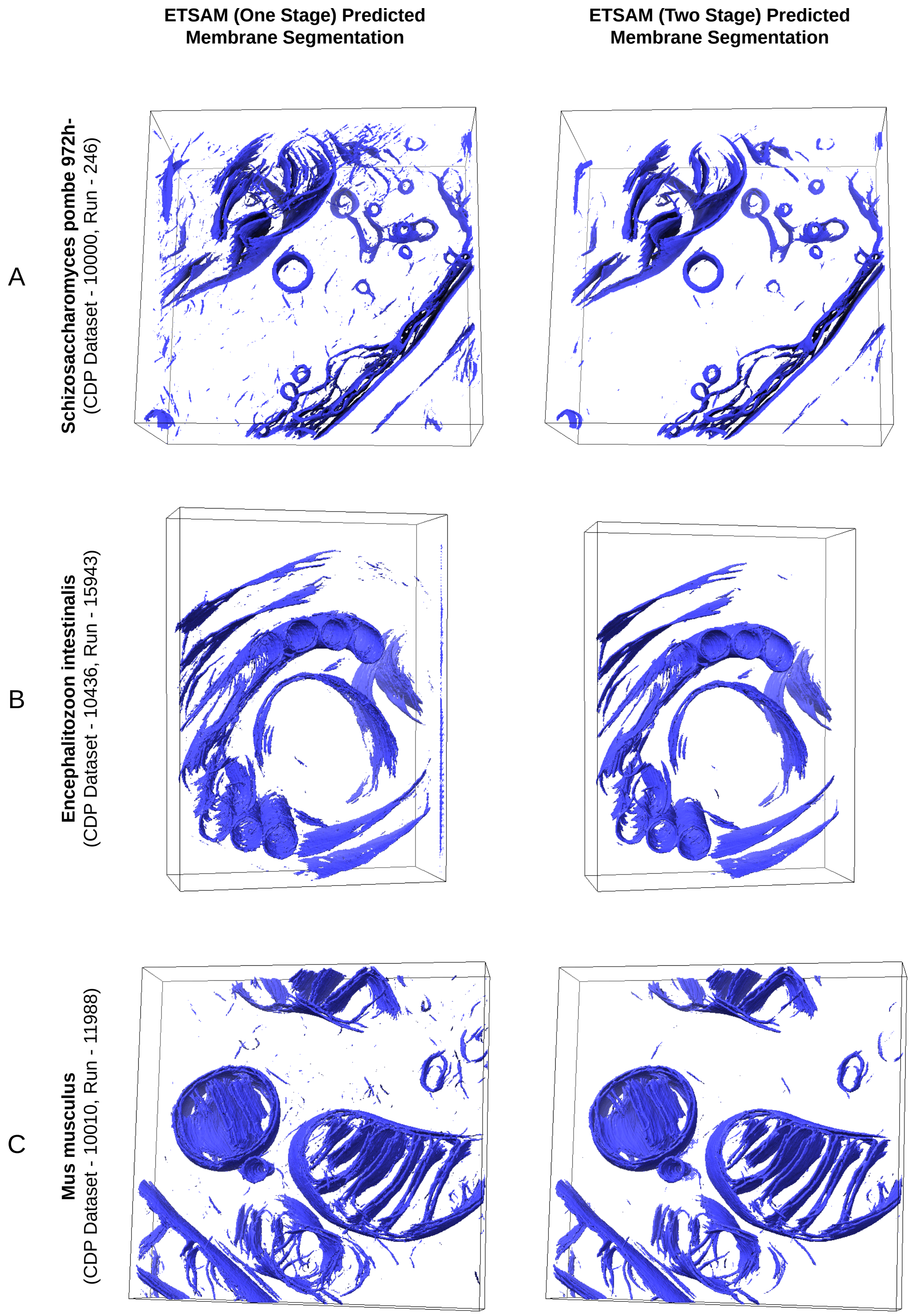

Fig. S3. Comparison between ETSAM (one stage) and ETSAM (two stage) predicted membrane segmentation on experimental tomograms of Schizosaccharomyces pombe 972h- (CDP Dataset - 10000, Run - 246), Encephalitozoon intestinalis (CDP Dataset - 10436, Run - 15943) and Mus musculus cells (CDP Dataset - 10010, Run - 11988).

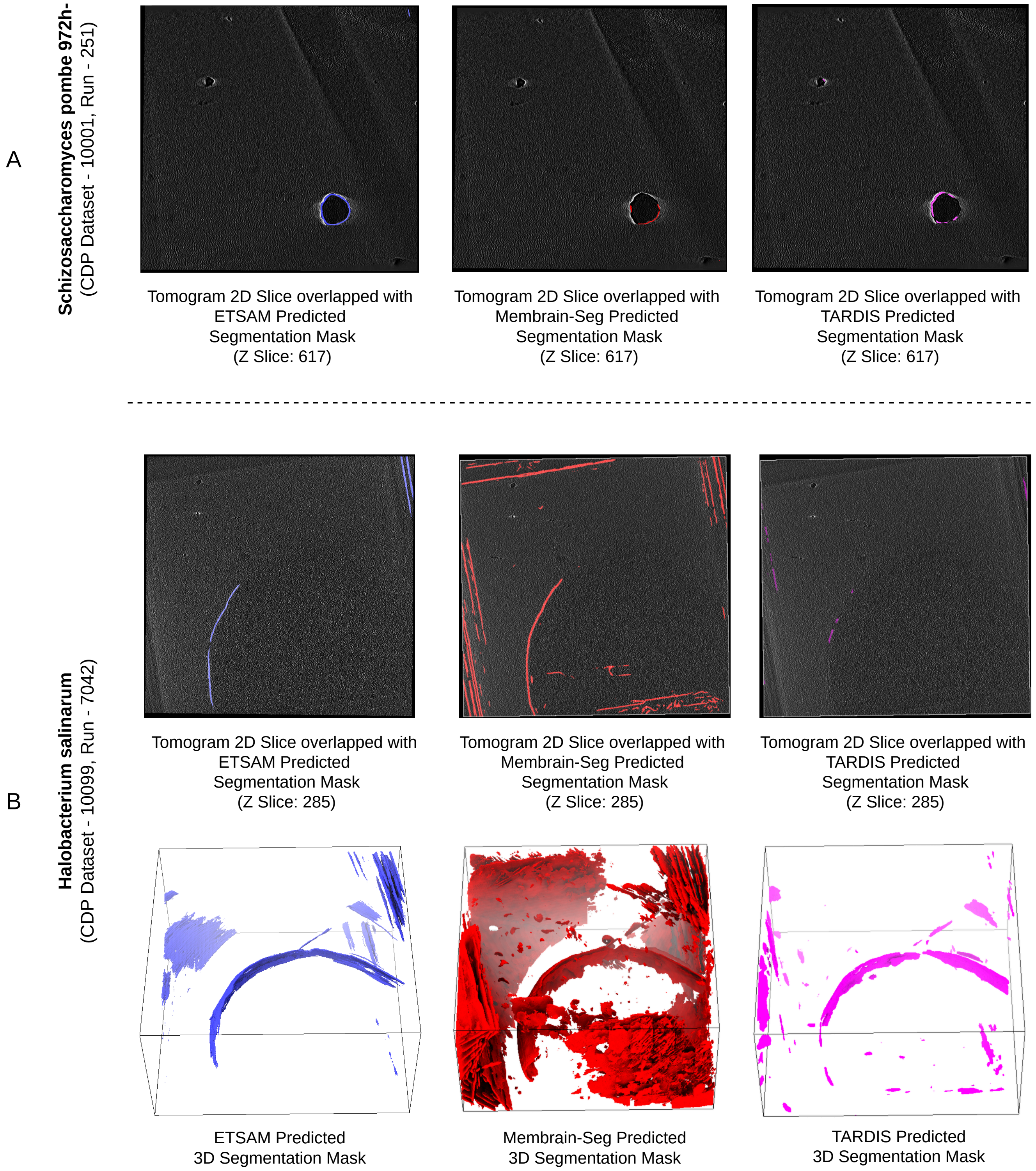

Fig. S4. Examples of showing the limitations of deep learning methods in handling noise artifacts. (A) shows membrane-like artifacts being predicted as a membrane region by ETSAM (blue), Membrain-Seg (red), and TARDIS (pink) in the bottom right of the tomogram slice of Schizosaccharomyces pombe 972h- cell (CDP Dataset - 10001, Run - 251). (B) compares border artifacts being predicted as membrane regions to various extents by ETSAM (blue), Membrain-Seg (red), and TARDIS (pink) in the tomogram of the Halobacterium salinarum cell (CDP Dataset - 10099, Run - 7042).
